# Divergent *Cotton leaf curl Multan betasatellite* and three different alphasatellite species associated with cotton leaf curl disease outbreak in Northwest India

**DOI:** 10.1371/journal.pone.0313844

**Published:** 2025-01-09

**Authors:** Kajal Kumar Biswas, Nenavath Balram, Marimuthu Elangovan, Supratik Palchoudhury, Utpal Kumar Bhattacharyya, Halima Khatoon, Shilpi Aggarwal, Shruti Godara, Pradeep Kumar, Satish Kumar Sain, Rupesh Arora, Sibnarayan Datta

**Affiliations:** 1 Division of Plant Pathology, Advanced Centre for Plant Virology, ICAR-Indian Agricultural Research Institute, New Delhi, India; 2 Regional Agricultural Research Station, PJTSAU, Jagtial, Telangana, India; 3 ICAR-Krishi Vigyan Kendra, Longding Kanubari, Arunachal Pradesh, India; 4 Forest Research Institute, Dehradun, Uttarakhand, India; 5 Agricultural Research Station, Swami Keshwanand Rajasthan Agriculture University, Sri Ganganagar, Rajasthan, India; 6 ICAR-Central Institute for Cotton Research, Regional Station, Sirsa, Haryana, India; 7 Regional Research Station, Punjab Agricultural University, Bhatinda, Punjab, India; 8 Entomology & Biothreat Management Division, Defense Research Laboratory (DRL-DRDO), Tezpur, Assam, India; Sakarya Uygulamali Bilimler Universitesi, TÜRKIYE

## Abstract

Cotton leaf curl disease (CLCuD) is a major constraint for production of cotton (*Gossypium* sp.) in Northwest India. CLCuD is caused by a monopartite, circular ssDNA virus belonging to the genus *Begomovirus* in association with betasatellites and alphasatellites, and ttransmitted by a whitefly vector (*Bemisia tabaci*). To explore the genetic variability in betasatellites and alphasatellite associated with the CLCuD-begomovirus complex in Northwest India. A survey was conducted for successive three years of 2014 to 2016 and twig samples from symptomatic and healthy cotton plants randomly were collected. Total plant DNAs were isolated, subjected to rolling circle amplification (RCA), cloning and sequencing. Full-length genome of 12 betasatellites and 13 alphasatellites, those were obtained in the present study, were analyzed. Sequence analysis showed that all the present betasatellites shared 85–99 percent nucleotide identity (PNI) among themselves and 84–95 PNI with other members of *Cotton leaf curl Multan betasatellite* (CLCuMB) and fell into one genogroup along with CLCuMB. But in close observation the present betasatellites clustered into two phylogenetic subgroups under single CLCuMB. The present alphasatellites showed 72–100 PNI among themselves and fell under three alphasatellite species, *Gossypium Darwinii symptomless alphasatellite* (GDarSLA), *Cotton leaf curl Multan alphasatellite* (CLCuMA) and *Cotton leaf curl Burewala alphasatellite* (CLCuBuA). In the recombination analysis, all the present betasatellites and alphasatellites were found to be recombinants involving intra species recombination in betasatellite, and interspecies recombination in alphasatellite species. The present study indicated that the betasatellite and alphasatellite molecules associated with CLCuD-begomovirus complex in Northwest India are genetically diverse.

## Introduction

Cotton (*Gossypium* spp.) is one of the major commercial crops cultivated in India and accounts to produce 362.18 million bales (2021–22), translating to around 25% of the total global production. It plays a major role in sustaining the livelihood of about 6 million farmers and 50 million people associated with textile industries. Even though, India ranks1^st^ in cotton acreage with 120.69 Lha (36% of the world cotton growing area), it ranks 38^th^ with a yield of 510 kg/ha. In India, 4 species of cotton, namely *G*. *arboretum*, *G*. *herbaceum* (Asian cotton), *G*. *barbadense* (Egyptian cotton), and *G*. *hirsutum* (American Upland cotton) are cultivated, *G*. *hirsutum* is grown in more than 90% of the cotton growing areas, of which 94% area is under the cultivation of hybrid Bt-cotton [1].

Cotton leaf curl disease (CLCuD) is a major constraint for cultivation of cotton (*G*. *hirsutum*) in the Indian subcontinent [2–6]. The entire cotton growing area of 1.1 million Mha in three States, Haryana, Punjab, and Rajasthan in Northwest India, are affected by CLCuD, causing enormous loss in crop yield [7,8]. The disease is characterized by leaf curling, dark green veins, and vein thickening and very often by producing small leaf-like structure (called enation) at the lower abaxial surface of the leaf. Cotton plants infected at an early stage shows stunted growth leading to huge crop losses [8,9]. Most of the cotton cultivars, including hybrid Bt-cotton, cultivated in Northwest India are highly susceptible to CLCuD by showing an array of disease severity, ranging from 10.8 to 77.5% loss [7–10].

In India, CLCuD was first reported from an experimental farm of Indian Agricultural Research Institute (IARI), New Delhi during 1989 and in the farmer’s field of Sri Ganganagar, Rajasthan, during 1993 [11,12]. Consequently, this disease has spread to almost all the cotton growing areas of Haryana, Punjab, and Rajasthan during 1992–1997. Within a relatively short period of time, the disease has established itself as a serious constraint for cotton cultivation in Northwest India during 2009–2010 [6]. CLCuD appeared as an epidemic during 2013 when 100% disease incidence with approximately 40% yield loss was documented [8].

CLCuD is caused by a monopartite circular single stranded DNA virus (~2.7 kb) belonging to the genus *Begomovirus*, and is transmitted by an insect vector, whitefly (*Bemisia tabaci*) [2,4]. Till date, nine distinct monopartite begomoviruses have been reported to be associated with CLCuD in the Indian subcontinent [6,13]. Of these, the *Cotton leaf curl Alabad virus* (CLCuAlV), *Cotton leaf curl Kokhran virus* (CLCuKoV) and *Cotton leaf curl Multan virus* (CLCuMuV) are most widely distributed in the Indian subcontinent [5,6,13,14]. Presently, CLCuMuV-Rajasthan (CLCuMuV-Ra) and CLCuKoV-Burewala (CLCuKoV-Bu) are reported to be prevalent strains in Northwest India [3,6,14–16], while CLCuMuV-Pakistan (CLCuMuV-PK), CLCuMuV-Ra and CLCuKoV-Shadadpur (CLCuKoV-Sha) strains are prevalent in adjacent cotton growing regions of Pakistan [17].

Monopartite begomoviruses are associated with circular ssDNA satellite molecules known as betasatellite (~1.3 kb) and alphasatellite (~1.4 kb) to cause CLCuD. Betasatellites contain three conserved features- the satellite conserved region (SCR), the βC1 gene, and an Adenine-rich (A-rich) region [18,19]. The βC1 gene determines pathogenicity, suppress post transcriptional gene silencing (PTGS), and *in planta* virus movement [20]. Alphasatellite is another diverse group of self-replicating satellite like circular ssDNA molecule associated with the CLCuD-begomovirus-betasatellite complex. It has three conserved regions- (i) a nanonucleotide (TAGTATT/AC) stem-loop sequence, similar to nanoviruses, having origin where Rep cleaves DNA to start replication, (ii) an ORF of Rep protein (~ 36.6 kDa), having up to 315 amino acids (aa), and (iii) an A-rich region of ~200 nt in length [21–23]. These satellite molecules are having direct or indirect functions while associating with their helper viruses. Betasatellite molecules are responsible for disease severity and believed to be involved in symptom expression and alphasatellite molecules believed to have role in suppression of post transcription gene silencing (PTGS) and attenuation of symptoms. Previously, single betasatellite species, the *Cotton leaf curl Multan betasatellite* (CLCuMB) has been detected in the CLCuD-begomovirus complex in the Indian subcontinent [5,6,14–17]. Zubair et al., [24] reported CLCuMB is the most important component of cotton leaf curl disease complex and identified three distinct CLCuMB in Pakistan.

Several numbers of alphasatellite species, *Cotton leaf curl Multan alphasatellite* (CLCuMA), *Cotton leaf curl Lucknow alphasatellite* (CLCuLucA), *Croton yellow vein mottle alphasatellite* (CrYVMoA), *Gossypium Darwinii symptomless alphasatellite* (GDarSLA) and *Gossypium mustilinum symptomless alphasatellite* (GMusSLA) have been reported in CLCuD-begomovirus complex in the Indian subcontinent [5,6,14,15,24–26].

Genetic variability of CLCuD-begomovirus and its associated betasatellite and alphasatellite complex plays an important role in disease outbreak and crop productivity in cotton growing regions in Indian-subcontinent. Begomoviruses and its satellite molecules associated with this disease are undergone rapid recombination that results in evolution of new variants to escape the host resistance. The knowledge about genetic diversity of satellite molecules associated with the disease complex is necessary to formulate the strategy to manage the CLCuD in the present scenario. Thus, the present study was carried out to elucidate genetic diversity, evolution and distribution of new variants of these satellite molecules in cotton growing areas in Northwest India.

## Materials and methods

### Survey of CLCuD in Northwest India and estimation of disease incidence and percent disease index

**Fig 1:**
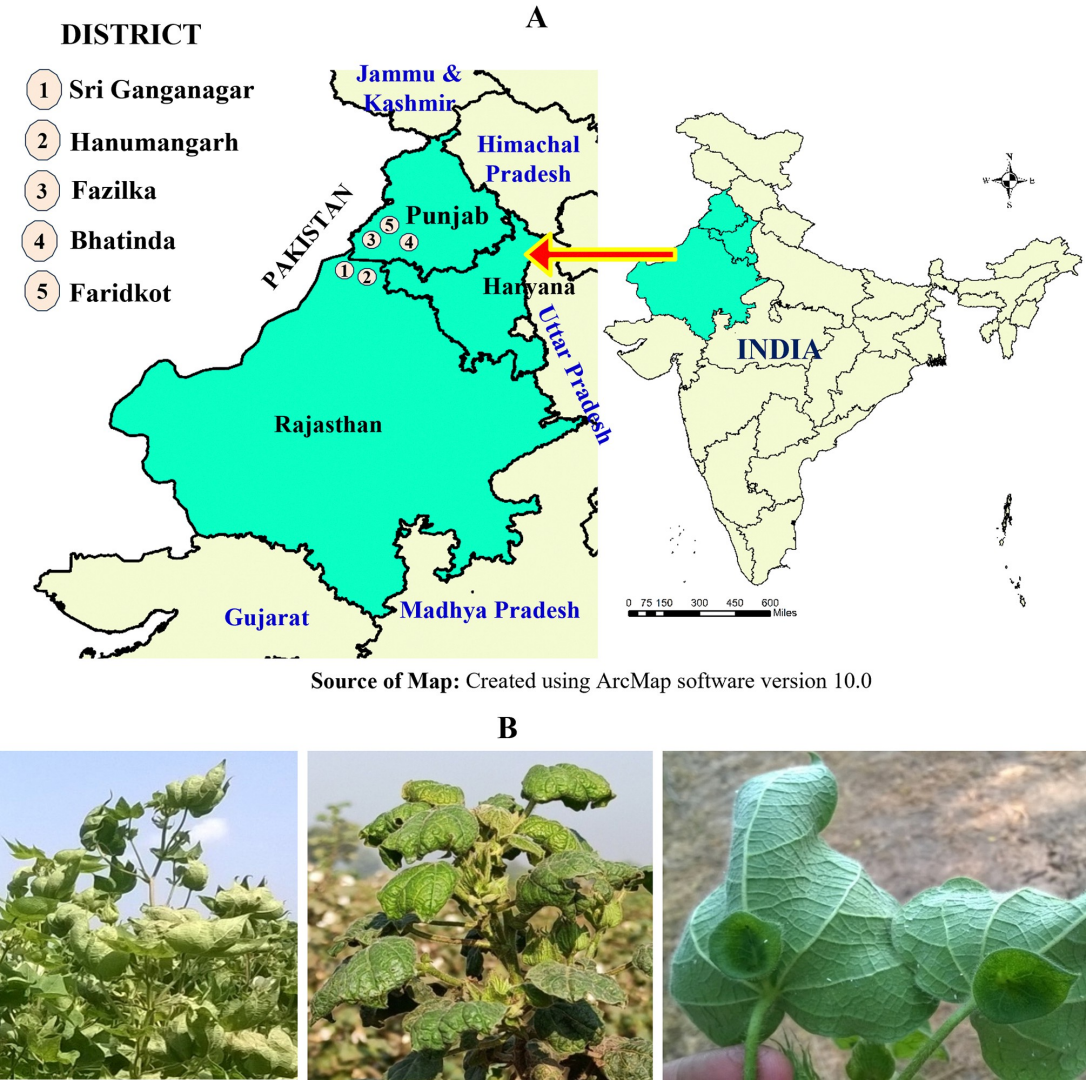
Survey of CLCuD symptoms in cotton fields of Northwest India. A. Cotton growing states in Northwest India surveyed, B. CLCuD symptoms in cotton of farmer’s field of Northwest India.

A survey was conducted to study CLCuD in cotton growing districts, namely Sri Ganganagar and Hanumangarh in the state of Rajasthan; Fazilka, Bathinda, and Faridkot in the state of Punjab of Northwest India (Fig 1) for the three successive years from 2014 to 2016. All the five districts selected in the present study shared border with Pakistan. About 12–28 fields for 2014, 13–66 for 2015 and 15–54 for 2016 from each of the districts were surveyed. Most of the farmers cultivated commercially available cotton hybrids. Disease incidence was estimated using the standard method {(No. of plants infected divided by No. of plants randomly taken in a particular block) multiplied by 100}. The percent disease index (PDI) was calculated using the standard method with the formula mentioned below:

$$PDI=\sum_{r=1}^{n}\frac{RF}{N}\times \frac{100}{G}$$

Where, R-Disease grade rating; F-Frequency of plants showing disease rating; N-Total number of plants observed for the presence of CLCuD symptoms; G-Highest grade of disease rating grade. The standard disease scales for rating of CLCuD used popularly in India developed earlier [27] were followed in the present study: 0-Absence of any symptoms; 1-Thickening of small veins, a few, scattered on one or few leaves of plant; 2-Thickening of small group of veins, no leaf curling, no reduction in leaf size and no reduction of boll setting; 3-Thickening of all the veins, minor leaf curling, leaf enation, deformity of internodes with minor reduction in leaf size but no reduction in boll setting; 4-Severe vein thickening, moderate leaf curling, leaf enation, minor deformity of internodes and minor reduction in leaf size and minor reduction of boll setting; 5-Severe vein thickening, moderate leaf curling, leaf enation, and moderate deformity of internodes with moderate reduction in leaf size and moderate reduction in boll setting followed by moderate plant stunting; 6-Severe vein thickening, leaf curling, reduction in leaf size, leaf enation, deformed internodes and severe stunting of plant with no or few boll setting. Cotton plants exhibiting typical CLCuD symptoms in the field were selected randomly.

### Collection of samples and extraction of total plant DNA

Infected twigs along with leaves were collected, kept in moist polythene bags, and brought to the laboratory for further processing. Two to four twig samples from all five districts surveyed were collected as representative samples from these districts (Table 1). Total DNA from symptomatic leaf samples from field and healthy cotton plants maintained in insect-proof greenhouse were extracted using CTAB method [28]. Total DNA was eluted in 100 μL pre-warmed buffer BET (70˚C) in a 1.5 ml micro centrifuge tube. The quality of DNA was checked by micro volume Spectrophotometer (NanoDrop, Thermo Fisher Scientific Inc, model No. ND1000) and visualized by electrophoresis on a 1% agarose gel in 1X TAE buffer.

**Table 1:** Features of complete betasatellite and alphasatellite molecules associated with CLCuD-begomoviruses infecting cotton in Northwest India.

S.N.	CLCuD infected samples	Location and year of collection	Symptom’s character	Putative CLCuD–begomovirus based on CP gene (Acc. No)	Satellite molecule	Accession Number	Size (nt)	ORF- βC1 (coordinates/ nt/aa)	ORF- Rep (coordinates/ nt/aa)
1	ARSB-15-1	Bhatinda (Punjab), 2015	VT, UC, LE (Var. H-1236)	CLCuKoV (MF462117)	CLCuMB	KY523512*	1358	194-550/357/118	-
					CLCuMuA	MF141732	1371	-	77-1018/942/313
2	ARSB-15-7	Bhatinda (Punjab), 2015	VT, UC, LE (Var. F-846)	CLCuKoV (MF462118)	CLCuMB	KY523513	1357	194-550/357/118	-
					CLCuMuA	MF141733	1372	-	77-1018/942/313
3	ARSF-15-1	Farikot (Punjab), 2015	VT, DC (Var. HS-6)	CLCuKoV (MF462119)	CLCuMB	KY523514	1358	194-550/357/118	-
					CLCuMuA	MF141734	1371	-	77-1018/942/313
4	ARSF-15-7	Farikot (Punjab), 2015	VT, DC (Hyb. Ankur 3028)	CLCuMuV (MF462120)	CLCuMB	KY523515	1458	194-550/357/118	-
					CLCuMuA	MF141735	1374	-	77-1018/942/313
5	Fz -15-1	Fazilka (Punjab), 2015	VT, DC, LE (Hyb. MRC 70)	CLCuMuV (MF462121)	CLCuMB	KY523516	1357	195-551/357/118	-
					CLCuBuA	MF141736	1368	-	77-1024/948/315
6	Fz-15-7	Fazilka (Punjab), 2016	VT, DC, UC, LE (Hyb. RCH 650)	CLCuMuV (MF462113)	CLCuMB	KY523512*	1358	194-550/357/118	-
					CLCuBuA	MF141737	1368	-	77-1024/948/315
7	Fz -15-10	Fazilka (Punjab), 2015	VT, DC (Hyb. RCH 650)	CLCuKoV (MF462122)	CLCuMB	KY523517	1357	195-551/357/118	-
					GDarSLA	MF141738	1368	-	70-1017/948/315
8	Fz-16-7	Fazilka (Punjab), 2016	VT, DC, UC, LE (Hyb. RCH 650)	CLCuKoV (MF667821)	CLCuMB	KY523512*	1358	194-550/357/118	-
					CLCuBuA	MF141739	1366	-	77-1024/948/315
9	Hmg-14-1	Hanumangarh (Rajasthan), 2014	VT, DC, LE, (Hyb. Bio 6488)	CLCuKoV (MF462123)	CLCuMB	KY523518	1354	194-550/357/118	-
					CLCuMuA	MF141740	1366	-	77-1018/942/313
10	Hmg-15-6	Hanumangarh (Rajasthan), 2015	VT, UC (Hyb. RCH 650)	CLCuMuV (MF462124)	CLCuMB	KY523519	1355	195-551/357/118	-
					GDarSLA	MF141741	1366	-	70-1017/948/315
11	Hmg-16-1	Hanumangarh (Rajasthan), 2016	VT, DC, LE, (Hyb. Bio 6488)	CLCuKoV (MF667822)	CLCuMB	MF141730	1355	194-550/357/118	-
					GDarSLA	MF141743*	1374	-	70-1017/948/315
12	SG-14-23	Sri Ganganagar (Rajasthan), 2014	VT, DC, UC, (Var. RST-9)	CLCuKoV (MF462125)	CLCuMB	KY523520	1359	194-550/357/118	-
					GDarSLA	MF141742	1373	-	70-1017/948/315
13	SG-15-11	Sri Ganganagar (Rajasthan), 2015	VT, DC, UC, LE (Hyb. NCS855)	CLCuKoV (MF462126)	CLCuMB	KY523521	1358	194-550/357/118	-
					GDarSLA	MF141743*	1374	-	70-1017/948/315
14	SG-16-5	Sri Ganganagar (Rajasthan), 2016	VT, DC, UC, LE (Hyb. RCH 650)	CLCuMuV (MF667824)	CLCuMB	MF141731	1355	194-550/357/118	-
					GDarSLA	MF141744	1374	-	70-1017/948/315

VT: Vein thickening, UC: Upward curling, DC: Downward curling,LE: Leaf enation, Var.: Variety (cotton), Hyb: Hybrid (cotton); * The complete nucleotide sequences of betasatellites obtained from isolates ARSB-15-1, Fz-15-7 and Fz-16-7 and complete sequence of alphasatellites from isolates Hmg-16-1 and SG-1511were cent percent identical.

### Infectivity test of CLCuD through whitefly inoculation and its confirmation by PCR

To assess the infectivity of CLCuD-begomoviruses isolated from infected cotton field of Northwest India, a whitefly mediated virus inoculation method using susceptible cotton plant developed earlier [12] was used in the present study. For confirmation of CLCuD-begomovirus infection in whitefly mediated virus inoculated cotton plants of greenhouse, the total plant DNAs were isolated. The complete CP and βC1 gene sequences were amplified using the specific primes of (C3F:5’-AATTATGTCGAAGCGAGCTG-3’/G1R:5’-TAATATCAATTCGTTACAGAG-3’) targeting CP gene of CLCuD-begomoviruses. PCR was performed in an automated thermal cycler (Bio-Rad, Germany) with the following parameters: one cycle of initial denaturation at 95°C for 5 min, followed by 30 amplification cycles of denaturation at 95°C for 50s, annealing at 54°C for 30s, extension at 72°C for 45s, and one cycle at 72°C for 10 min for final extension. Amplicons were analysed on a 1% agarose gel electrophoresis.

### Amplification and cloning of CP genes, full-length genome of betasatellites and alphasatellites associated with CLCuD-begomovirus

Complete CP gene of CLCuD begomovirus from CLCuD affected field collects of cotton plant samples were amplified through specific primes targeting CP gene, purified, cloned, sequenced, and analysed. Full-length genomes of betasatellite and alphasatellite molecules were amplified through rolling circle amplification (RCA) method, following method developed earlier [29]. The amplified product obtained through RCA was used as a template for amplification of full-length betasatellite and alphasatellite genome using universal primers, β01 and β02 [30] for betasatellite and DNA101 and DNA102 [31] for alphasatellite molecule. The PCR thermal profile was following: one cycle at 94°C for 4 min: 30 cycles at 94°C for 30s (denaturation), 52.0(alphasatellite)/54.5°C(betasatellite) for 30s (annealing), 72°C for 45s (extension) and one cycle at 72°C for 10 min (final extension). Amplicons (~1.4 kb) were analysed on a 1% agarose gel electrophoresis. PCR products were purified using PCR purification kit (Qiagen) and cloned into the T/A cloning vector, as described elsewhere [32]. Two clones of each isolate were sequenced using vector derived primers M13 Forward and M13 Reverse on an automatic sequencer (Chromous Biotech Pvt. Ltd, Bangalore, India). The consensus sequences were taken for further analysis.

### Sequence analysis, phylogenetic relationship and recombination analyses

DNA sequence electropherograms from satellite molecules were quality-checked and manually edited. Vector sequences were removed using the BioEdit version 7.1.3 [33]. Coding regions of the satellite DNAs were identified using the NCBI ORF finder (www.ncbi.nlm.nih.gov/orffinder/) and annotated. All the sequences were searched for similarities using BLASTn tools, and respective top 10–15 sequence matches available in the GenBank (https://www.ncbi.nlm.nih.gov) were downloaded. For phylogenetic analysis, sequences were aligned using ClustalW program [34], implemented in the MEGA11 program [35]. Phylogenetic trees were reconstructed using Neighbour-Joining (NJ) method [36] with 1000 bootstrap iterations using the MUSCLE algorithm. Percentage nucleotide identity (PNI) was determined, using the Sequence Demarcation Tool (SDT) version 1.2 [37]. For sequence analyses of present satellite molecules and previously reported satellite molecules associated with CLCuD-begomovirus in cotton and begomoviruses in other crops were retrieved from the GenBank.

Recombination detection program (RDP) version 4.6 was used by implementing eight algorithms BootScan, Chimera, Geneconv, Maxchi, RDP, SiScan, 3Seq and LARD. Default settings with Bonferroni-correction were used to predict potential recombination events at a P-value cut-off of 0.05 [38]. Each event was verified based on a breakpoint distribution plot, and results were compared against UPGMA phylogenetic trees produced with genetic regions from major and minor parents. Recombination events detected by more than four algorithms in betasatellite and alphasatellite sequences were considered true recombination events.

### Ethics statement

No permits are required for collecting cotton plants from the farmer’s field in cotton growing areas in Northwest India during survey.

## Results and discussion

**Fig 2:**
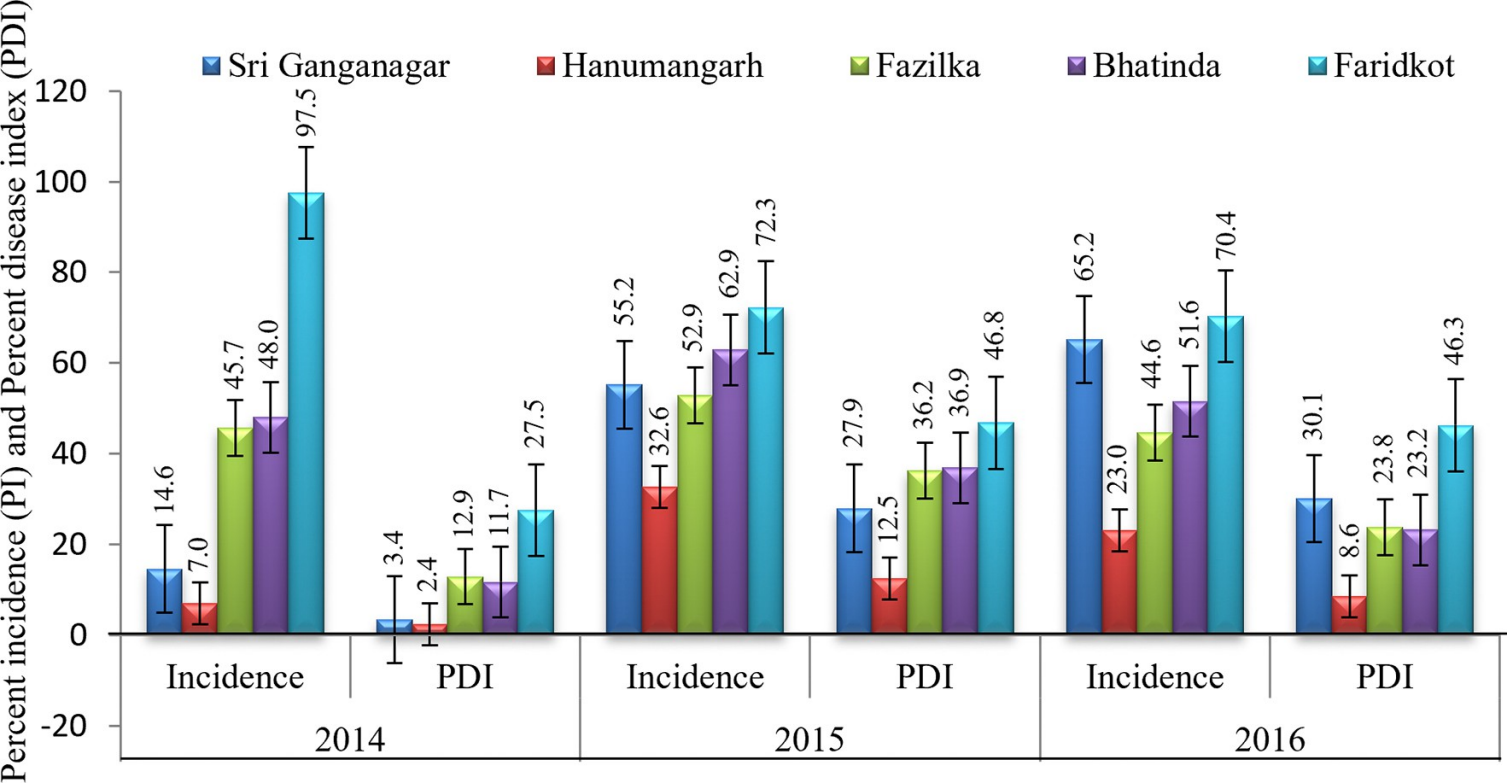
Percent disease incidence and PDI of CLCuD in different areas of Northwest India; 12–28 fields for 2014, 13–66 fields for 2015, 15–54 fields for 2016.

In the present study, different kinds of symptoms like downward and upward leaf curling, cupping of leaf, vein thickening, and sometimes enation underside of the leaves were observed in the cotton growing areas of Punjab and Rajasthan states of Northwest India during survey of 2014 to 2016. The overall CLCuD incidence of 7.0–97.5% with 2.4–27.5% disease severity (PDI) in 2014, incidence of 32.64–72.28% with 12.53–46.82% disease severity in 2015, and incidence of 23.01–70.35% with 8.61–46.27% disease severity in 2016 were recorded (S1 Table). The higher CLCuD incidence ranging from 70.4–97.5% with 27.5–46.8% disease severity was recorded in Faridkot district of Punjab in all the three years. The lower disease incidence ranging from 7.0–23.0% with 2.4–12.5% severity in Hanumangarh district of Rajasthan in all the three years. The CLCuD incidence (44.6–52.9%) in Fazilka district of Punjab was constant as compared to other districts for the three years of survey (Fig 2).

### Confirmation of CLCuD-begomoviruses associated with CLCuD in Northwest India

For confirmation of the CLCuD-begomoviruses, transmission of the CLCuD through whitefly was studied using susceptible cotton cv. RST-9 in a greenhouse. CLCuD-begomovirus infected cotton samples randomly collected from five cotton growing areas, Hanumangarh (Hmg-15-6), Bhatinda (ASRB-15-1), Faridkot (ARSF-15-1), Fazilka (Fz-15-1) and Sri Ganganagar (SG-15-11) of Northwest India were used as source of inoculums for whitefly transmission (Table 2). The whitefly inoculated cotton plant induced typical CLCuD symptoms of downward/upward leaf curling, vein thickening and leaf enation within 15–25 DAI with the transmission efficiency of 70–100% (Table 2). These findings conclude that whiteflies are efficiently transmitting the CLCuD-begomovirus in cotton plants in Northwest India.

**Table 2:** Infectivity test of CLCuD field isolates of Northwest India through whitefly inoculation in greenhouse condition.

Isolate	CLCuD source	Inoculated cultivar (in greenhouse)	Days taken to appear symptom after inoculation	No. pl infected/ No. of pl inoculated (%)	Symptoms induced	Detection CLCuD-begomovirus by amplification of CP gene through PCR
ASRB-15-1	Bhatinda (Punjab)	RST-9	17–20	7/10 (70)	DC, UC, VT	√
ARSF-15-1	Farikot (Punjab)	RST-9	16–25	9/12 (75)	UC, VT	√
Fz-15-1	Fazilka (Punjab)	RST-9	15–21	7/10 (70)	UC, VT	√
Hmg-15-6	Hanumangarh (Rajasthan)	RST-9	16–21	8/10 (80)	LE, DC	√
SG-15-11	Sri Ganganagar (Rajasthan)	RST-9	16–21	10/10 (100)	DC, LE, UC	√

DC: Downward Curling; UC: Upward Curling; VT: Vein Thickening; LE: Leaf Enation.

**Fig 3:**
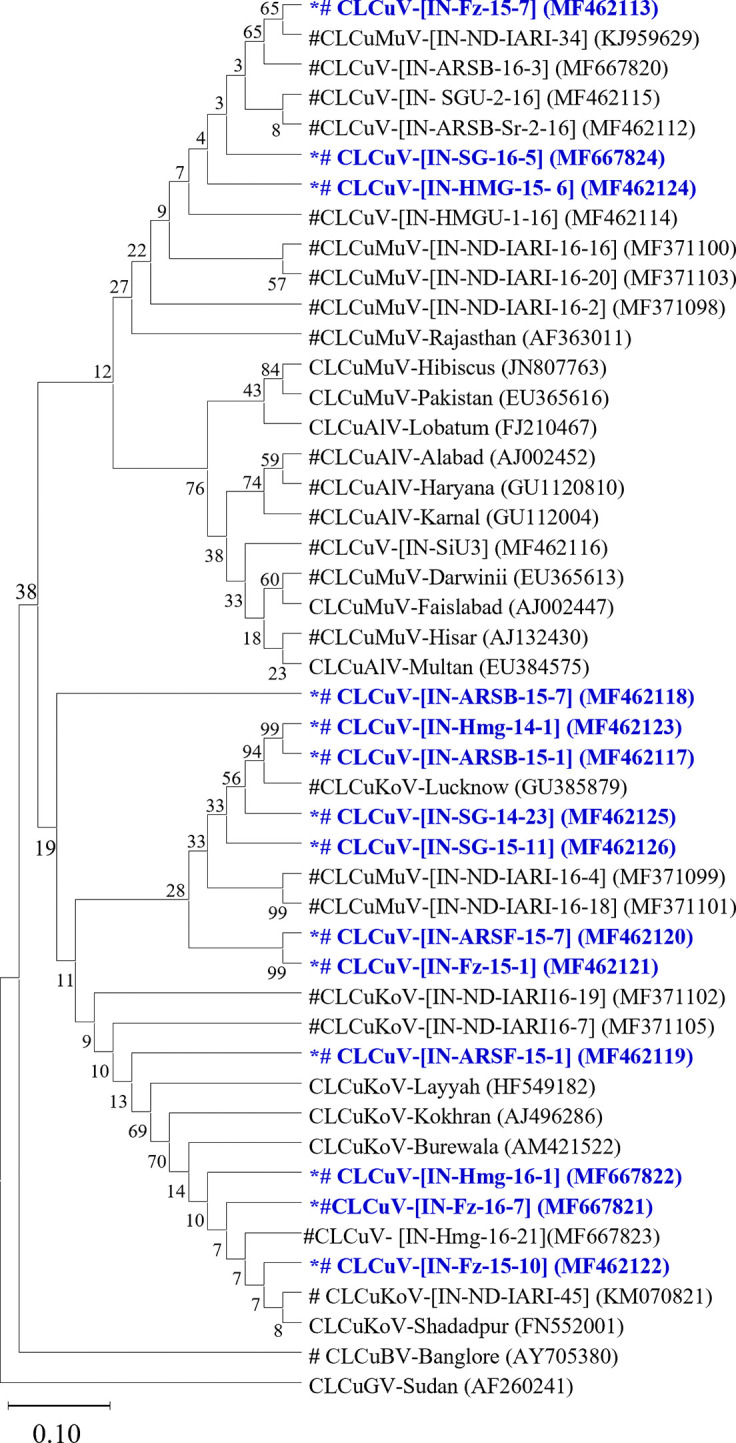
Phylogenetic relationships based on sequence analysis of complete CP gene of the CLCuD-begomoviruses those are associated with the present betasatellites and alphasatellite molecules, and other CLCuD-begomoviruses. The trees were generated using the NJ method in MEGA 11. The percentage of replicate trees in which the associated taxa clustered together in the bootstrap test (1,000 replicates) is shown next to the branches. The CP gene sequences generated in the present study are represented by asterisk. # represents Indian CLCuD begomovirus isolates.

To corroborate the presence of begomovirus associated with CLCuD, the complete CP genes of the 14 CLCuD-begomovirus genomes were amplified, cloned, and sequenced (Table 1). Nucleotide sequence analysis showed that the present CLCuD-begomovirus isolates shared 89–100 PNI among themselves and phylogenetic analysis showed that the present 11 CLCuD isolates were tentatively CLCuKoV, and other three were CLCuMuV species (Fig 3). Although, previously, Bhattacharya et al., reported that the pattern of phylogenetic trees constructed using the complete genomes and complete CP genes of CLCuD-begomovirus species were nearly similar. These data conclude that the satellite molecules, those were used in the present study, are associated with the CLCuD-begomovirus complex in cotton growing areas of Northwest India.

### Typical betasatellites associated with CLCuD-begomovirus complex in Northwest India

Complete genomes of 14 present betasatellites associated with CLCuD-begomoviruses in Northwest India were amplified, cloned, sequenced and analysed. As present betasatellites ARSB-15-1, Fz-15-7, and Fz-16-7 showed 100 PNI, the betasatellite ARSB-15-1 was selected for further analysis. Genome size of the present betasatellites ranged from 1354 to 1358 nt (Table 1), containing three conserved features; (i) satellite conserved region (SCR), (ii) single ORF (βC1), and (iii) an adenine (A) rich (A-rich) region. All the present betasatellites had a ~357 nt βC1 gene in the complementary sense, typical to the βC1 gene of other betasatellite molecules associated with CLCuD-begomoviruses (Table 1). Pairwise sequence identity analysis of nucleotide (nt) and deduced amino acids (aa) showed that βC1 genes of the present betasatellites shared 99–100 PNI and 94–100 per cent aa identity among themselves; and 94–100 PNI and 70–99 per cent aa identity with βC1 genes of other betasatellites associated with CLCuD-begomoviruses. Length of the satellite conserved regions **(**SCR) of all the present betasatellites ranged from 129 to 131 nts (S2 Table). At the 3’ end of the betasatellite genome, a predicted hairpin structure containing the canonical nanonucleotide sequence (TAATATTAC) within the loop was observed. Sequences of the hairpin stem of all the present betasatellites were found to be identical. A-rich regions of the present betasatellites had the typical length of 119–130 nt having adenine (A) content between 56.4 and 60.7%, as compared to overall adenine (A) content ranging from 28–38% in the betasatellite genome. This region has repeated blocks of up to eight consecutive adenine residues (S3 Table).

### Single CLCuMB species and its variants associated with CLCuD-begomovirus complex in Northwest India

Complete genome of the present 12 betasatellites were analysed and compared with other betasatellite molecules. The present betasatellites shared 87–99 PNI among themselves, and 84–95 PNI with the members of *Cotton leaf curl Multan betasatellite* (CLCuMB) (Table 3) and 42–80 PNI with betasatellites associated with other begomoviruses. The present betasatellites showed 86–90 PNI with CLCuMB (GenBank Accession No. AJ298903), which is proposed as the type species by Adams et al. [39]. Based on the species demarcation criteria of ≥78 PNI, as proposed by Bridon et al. [18], the present betasatellites were members under CLCuMB, revealing that a single betasatellite species, CLCuMB is associated with CLCuD complex in Northwest India.

**Table 3:** Percent nucleotide identity matrix of complete genome of betasatellite associated with the present and other CLCuD-begomoviruses and other hosts infecting begomoviruses.

Betasatellite	Betasatellites of the present CLCuD-begomoviruses												Betasatellites of CLCuD-begomoviruses				Other host infecting betasatellites				
	1	2	3	4	5	6	7	8	9	10	11	12	13**	14	15	16	17	18	19	20	21
CLCuMB-[IN-ARSB-15-1B]–(KY523512) (1)	100	97	96	97	92	96	96	95	96	97	97	89	87	90–92	85–91	85–89	56	55	47	42	47
CLCuMB-[IN-ARSB-15-7B]–(KY523513) (2)		100	98	99	94	99	98	98	98	97	99	91	89	92–94	87–93	86–91	57	56	47	43	47
CLCuMB-[IN-ARSF-15-1B]–(KY523514) (3)			100	98	93	97	97	96	97	96	98	90	88	91–93	86–92	86–90	57	56	47	44	47
CLCuMB-[IN-ARSF-15-7B]–(KY523515) (4)				100	95	99	98	98	98	97	99	91	89	92–93	88–93	87–92	57	56	47	44	48
CLCuMB-[IN-Fz-15-1B]–(KY523516) (5)					100	95	94	94	94	93	95	87	86	89–90	84–89	84–88	56	55	47	43	48
CLCuMB-[IN-Fz-15-10B]–(KY523517) (6)						100	98	98	98	97	99	91	89	90–94	87–93	8792	57	56	47	43	48
CLCuMB-[IN-Hmg-14-1B]–(KY523518) (7)							100	97	97	96	98	91	89	90–94	87–94	87–91	57	56	48	43	48
CLCuMB-[IN-Hmg-15-6B]–(KY523519) (8)								100	97	96	98	92	89	90–93	88–93	88–93	57	56	48	44	48
CLCuMB-[IN-Hmg-16-1B]–(MF141731) (9)									100	96	99	91	89	90–93	87–93	86–91	57	56	47	43	48
CLCuMB-[IN-SG-14-23B]–(KY523520) (10)										100	97	89	88	91–92	85–91	85–90	56	55	47	42	46
CLCuMB-[IN-SG-15-11B]–(KY523521) (11)											100	91	87	93–94	88–94	87–92	57	56	47	44	48
CLCuMB-[IN-SG-16-5B]–(MF141730) (12)												100	90	88	87–90	87–95	55	57	45	43	48

13^**^: represents the Type member CLCuMB-(AJ298903), CLCuMB-[PK-Mul-U89-97](AJ298903); **14**: CLCuMB-[IN-02](AY083590),CLCuMB-[IN-04](AY795604), CLCuMB-[IN-Pun-Ma14_3–14](KT228325); **15**: CLCuMB-[PK-Mul-07](EU384580), CLCuMB-[IN-Pun-09](HQ257372), CLCuMB-[IN-Sir-Si14_1–14](KT228326),CLCuMB-[IN-SG-05](JF502375); **16**: CLCuMB-[PK-Mul-08](HE601938), CLCuMB-[IN_IARI_34–13](KM070822), CLCuMB-[PK-Tjam-06](HE601940), CLCuMB-[IN-Pun-05](DQ191161), CLCuMB-[PK-Veh-05] (AM084379), CLCuMB-[PK-Mul-07](EU384579), CLCuMB-[PK-Mul-06](AM712314); **Others 17**: RLCuB[In-Var-06](EF175734), **18**: BhYVB[IN-Var-08](HM590506), **19**: ChLCuB[IN-Mr-12](JX193616), **20**:ALCB-[Cam-Bue-09](NC_012557), **21:**PLCuB-[In:ND:03](NC004706).

**Fig 4:**
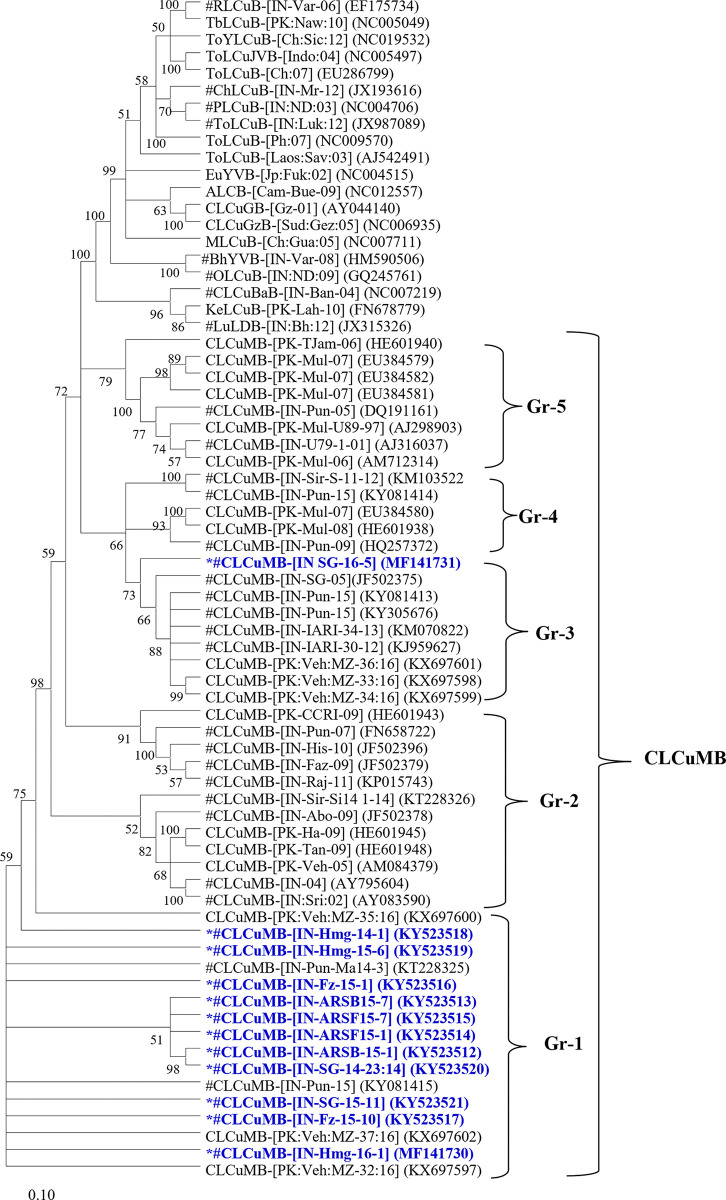
Phylogenetic relationships based on the complete genome of the present betasatellite molecules associated with CLCuD begomovirus complex in Northwest India with other betasatellite molecules in NCBI-GenBank. Phylogenetic tree was generated using the Neighbor-Joining (NJ) with 1000 bootstrap iterations in MEGA 11. The sequences generated in the present study are represented by asterisk. Groups of the CLCuMB demarcated in the right panel of the figure. # represents the betasatellites associated CLCuD begomoviruses from India.

**Fig 5:**
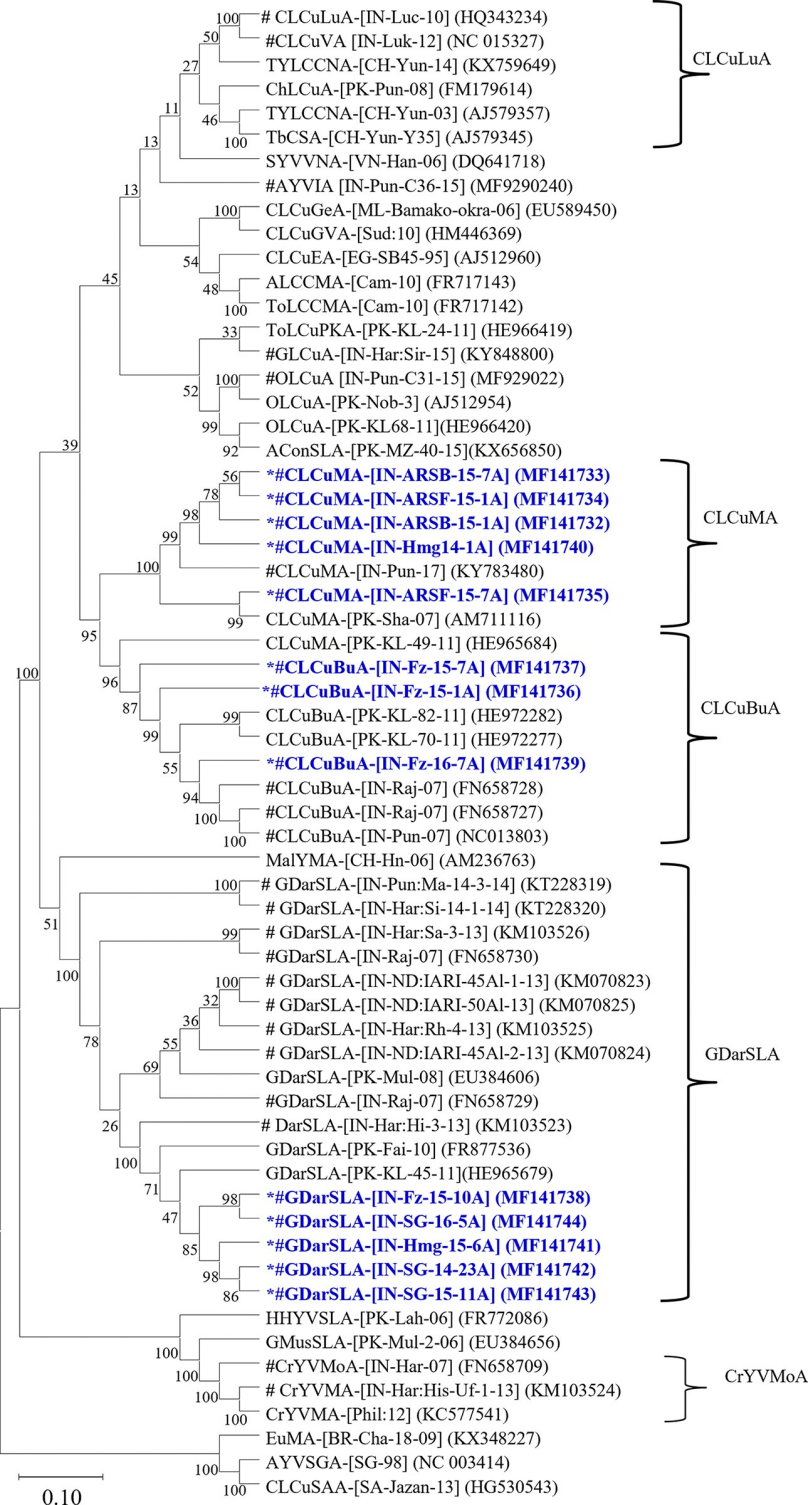
Phylogenetic relationships based on the complete genome of the present alphasatellite molecules with other alphasatellites available in NCBI-GenBank. The Phylogenetic tree was generated using the Neighbor-Joining (NJ) with 1000 bootstrap iterations in MEGA 11 software. The sequences generated in the present study are represented by asterisk. GDarSLA, CLCuMA, CLCuLuA, CLCuBuA and CrYVMA clades demarcated in the right panel of the figure. # represents the alphasatellites associated CLCuD begomoviruses from India.

In the Phylogenetic tree analysis, the present betasatellite clustered into one genogroup along with betasatellite type member CLCuMB (AJ298903) (Fig 4). If this species demarcation threshold of 91 PNI [39,40] is considered and closely examined all the betasatellite including the present betasatellite associated with CLCuD-begomoviruses could be divided into five betasatellite group/subgroup species; CLCuMB Gr-1 to Gr-5 (Fig 5). The present betasatellite fell into two group, Gr-1 and Gr-3, where betasatellite into Gr-1 and one betasatellite SB-16-5B into Gr-3. However, another subgroup Gr-2, 4 and 5 contains betasatellite reported earlier from CLCuD-begomovirus complex [6,14,15,41]. Then the results of the present study indicate that the betasatellite under CLCuMB associated with CLCuD-begomovirus in India are diverse, where the members belonging to subgroup Gr-1 are predominant. In sharp contrast, three betasatellite viz; CLCuMB_Bur_, CLCuMB_Veh_ and CLCuMB_Mul_ were identified from CLCuD-begomovirus complex from Pakistan [46].

### Recombination in CLCuMB associated with CLCuD-begomovirus complex in Northwest India

**Fig 6:**
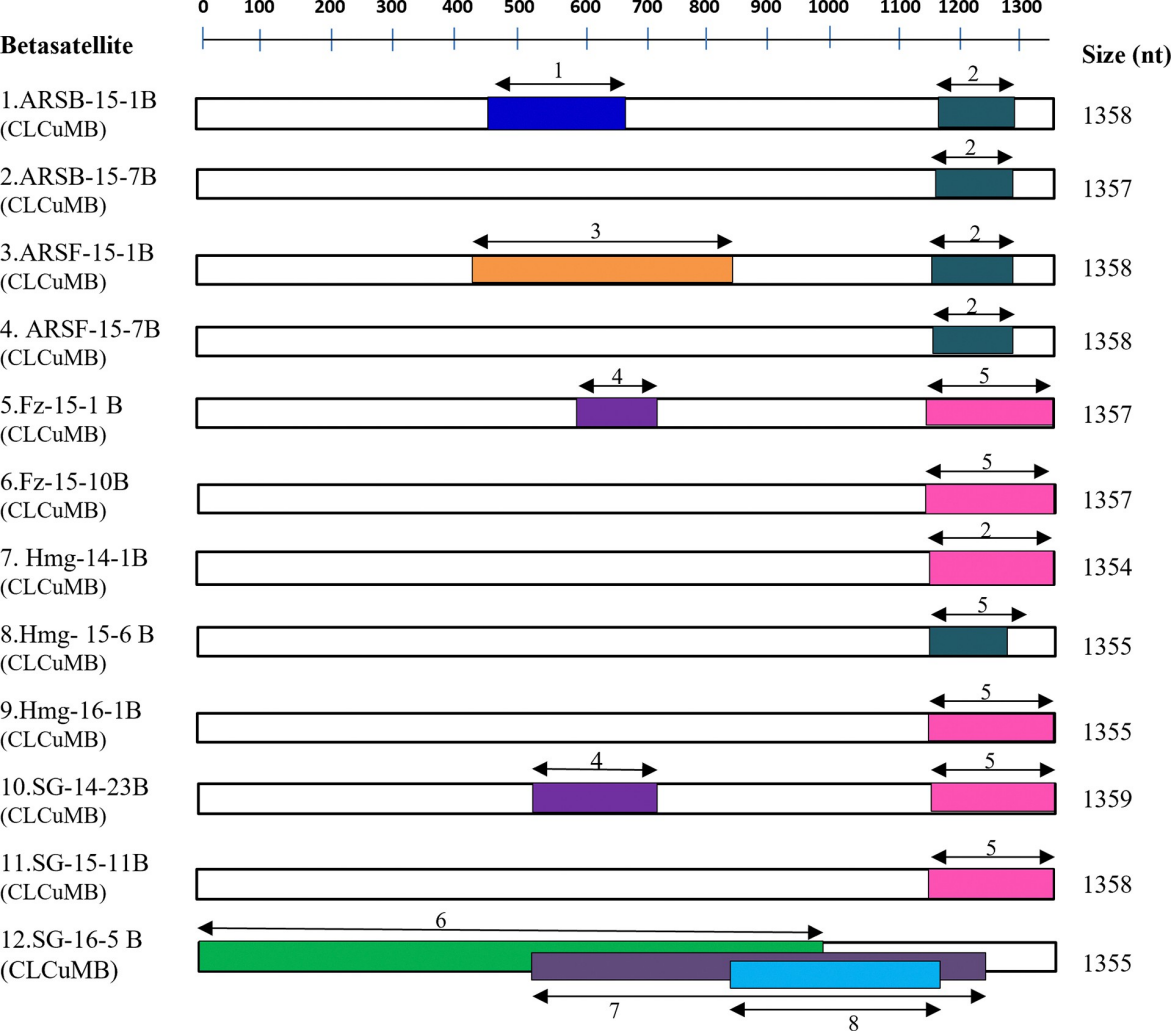
Recombination events identified in the present betasatellites associated with CLCuD-begomovirus. Genetic map of DNA of betasatellite molecule is shown at the top of the figure, recombinant fragments are represented by double arrow color bars along with minor parent involved in each recombination event represented by the bold number. Recombinant event with minor donor 1: CLCuMB-IN-Faz-09(JF502379); 2: PLCuB-[IN-ND-03](NC004706); 3: CLCuMB-[IN-Sir-14-1](KT228326); 4: CLCuMB-[IN-Faz-09](JF502379); 5: PLCuB-[IN-ND-03](NC004706); 6: CLCuMB-[PK-Ha-09](HE601945); 7: ToLCuB-[PH-07](NC009570); 8: CLCuMB-[PK-Veh-MZ-3616](KX697601). Shaded areas indicating the recombinant region in the genome of satellite molecule.

The recombination detection program RDP4 detected all the present betasatellites as recombinants and involving overall eight recombination break points (Table 4 and Fig 6). Three break points were detected in betasatellite SG-16-5B; two in betasatellites ASRB-15-1B, ARSF-15-1B, Fz-15-1B and SG-14-23B, and a common recombination event in the other present betasatellites were detected. All the present betasatellites showed recombination in the SCR region. The present finding indicates that this region of betasatellites is susceptible for recombination events. Previously, Zubair et al. reported of betasatellite that SCR is a recombination hotspot and this might be linked to the variability in the sequence length of SCR [46].

**Table 4:** Recombination analysis of betasatellites molecule associated with CLCuD-begomovirus genome using the RDP.

Betasatellite (Species)	Recombination site	Length (nt)	Event number	Major parent x Minor parent	Detected by method*	P Value (Max)**
1.ARSB-15-1B (CLCuMB)	440–663 (Partial βC1)	224	1	CLCuMB-IN-Hmg 14–1 (KY523518) x CLCuMB-IN-Faz-09 (JF502379)	R, G, M, C, S, 3S, B	2.8 x10^-4^
	1151–1298 (Partial SCR)	148	2	CLCuMB-[IN-U79_1–01] (EJ316037) x PLCuB[IN:ND:03] (NC004706)	R, G, M, C, S, 3S, B	3.1 x10^-5^
2.ARSB-15-7B (CLCuMB)	1149–1296 (Partial SCR)	148	2	CLCuMB-[IN-U79_1–01] (AJ316037) x PLCuB[IN:ND:03] (NC004706)	R, G, M, C, S, 3S, B	3.1 x10^-5^
3.ARSF-15-1B (CLCuMB)	439–832 (Partial βC1)	394	3	CLCuMB-[PK: Veh:MZ-37:16] (KX697602) x CLCuMB-[IN-Sir-14–1] (KT228326)	G, M, C, S, 3S, B	4.5 x10^-4^
	1150–1297 (Partial SCR)	148	2	CLCuMB-[IN-U79_1–01] (AJ316037) x PLCuB[IN:ND:03] (NC004706)	R, G, M, C, S, 3S, B	3.1 x10^-5^
4.ARSF-15-7B (CLCuMB)	1150–1297 (Partial SCR)	148	2	CLCuMB-[IN-U79_1–01] (AJ316037) x PLCuB[IN:ND:03] (NC004706)	R, G, M, C, S, 3S, B	3.1 x10^-5^
5.Fz-15-1B (CLCuMB)	591-715(Partial βC1)	125	4	CLCuMB-IN-Hmg 16–1 (MF141730) x CLCuMB-IN-Faz-09 (JF502379)	R, G, M, C, S, 3S	2.5 x10^-10^
	1148–0 (Major part of SCR)	210	5	CLCuMB-[IN-U79_1–01] (AJ316037) x PLCuB[IN:ND:03] (NC004706)	R, G, M, C, S, 3S, B	3.1 x10^-5^
6.Fz-15-10B (CLCuMB)	1148–0 (Major part of SCR)	210	5	CLCuMB-[IN-U79_1–01] (AJ316037) x PLCuB[IN:ND:03] (NC004706)	R, G, M, C, S, 3S, B	3.1 x10^-5^
7.Hmg-15-6B (CLCuMB)	1149–1275 (Partial SCR)	127	2	CLCuMB-[IN-U79_1–01] (AJ316037) x PLCuB[IN:ND:03] (NC004706)	R, G, M, C, S, 3S, B	3.1 x10^-5^
8.Hmg-14-1B (CLCuMB)	1149–0 (Major part of SCR)	207	5	CLCuMB-[IN-U79_1–01] (AJ316037) x PLCuB[IN:ND:03] (NC004706)	R, G, M, C, S, 3S, B	3.1 x10^-5^
9.Hmg-16-1B (CLCuMB)	1148–0 (Major part of SCR)	208	5	CLCuMB-[IN-U79_1–01] (AJ316037) x PLCuB[IN:ND:03] (NC004706)	R, G, M, C, S, 3S, B	3.1 x10^-5^
10.SG-14-23B (CLCuMB)	532–711 (Partial βC1)	180	4	CLCuMB-[IN-Hmg 14-1-14] (KY523518) x CLCuMB-[IN-Faz-09] (JF502379)	R, G, M, C, S, 3S, B	2.8 x10^-4^
	1150–0 (Major part of SCR)	210	5	CLCuMB-[IN-U79_1–01] (AJ316037) x PLCuB-[IN:ND:03] (NC004706)	R, G, M, C, S, 3S, B	3.1 x10^-5^
11.SG-15-11B (CLCuMB)	1149–0 (Major part of SCR)	210	5	CLCuMB-[IN-U79_1–01] (AJ316037) x PLCuB[IN:ND:03] (NC004706)	R, G, M, C, S, 3S, B	3.1 x10^-5^
12.SG-16-5B (CLCuMB)	0–989 (Complete βC1, partial SCR)	989	6	CLCuMB-[IN-U79_1–01] (AJ316037) x CLCuMB-[PK-Ha-09] (HE601945)	R, G, M, C, S, 3S, B	3.0 x10^-3^
	524–1237 (Partial βC1, partial SCR)	714	7	CLCuMB-[PK-Mul-08] (HE601938) x ToLCuB-[PH:07] (NC009570)	R, M, C, S, 3S	1.6 x10^-2^
	846–1166 (Partail A-Rich, SCR)	321	8	CLCuMB-[IN-Sir-S-11-12] (KM103522) x CLCuMB-[PK:Veh:MZ-3616] (KX697601)	R, G, M, S, 3S, B	2.6 x10^-4^

*: B: Bootscan, C: Chimera, G: Geneconv, M: Maxchi, R: RDP, Si:Siscan and 3-S: 3SEQ implemented in the RDP4 **: Highest acceptable P-value cut-off of 0.05 detected the evidences of recombination events among the sequences.

The betasatellite SG-16-5B showed multiple recombination event, three recombination break points covering most of the sequence, from positioned at 1to 1237 nts, encompassing complete βC1 gene, SCR, and A-rich genes. Interestingly, this betasatellite clustered distinctly from all other present betasatellites in the phylogenetic tree (Fig 6). All the present betasatellites showed intra-species recombination, where a present Indian betasatellite Hmg-14-1B and previously reported Indian betasatellite CLCuMB-[IN-U79-1-01] (AJ316037) were detected as major parent.

The first epidemic of CLCuD in Pakistan during the 1990s occurred due to the infection of multiple CLCuD-begomoviruses such as CLCuMuV, CLCuAlV and CLCuKoV-Ko, associated with a single betasatellite, CLCuMB [21,42,43]. The main reason for the epidemic is due appearance of new recombinant betasatellite variant CLCuMB_Veh_ occurring, recombination in the SCR region and this is different from the previously identified CLCuMB_Mul_ and recombinant CLCuMB_Bur_ variants. Subsequently, the second epidemic of CLCuD in Pakistan during 2000s was due to the emergence of the resistance breaking CLCuKoV-Bu, which was also associated with recombinant CLCuMB, where the SCR was replaced by a part of SCR sequence of distinct betasatellite, *Tomato leaf curl betasatellite* (ToLCB) [44,45]. In 2005, an increase in CLCuD in the Sindh province of Pakistan was shown to be associated with another recombinant CLCuMB containing a sequence fragment from ToLCB [46]. In 2015, a new recombinant CLCuMB was reported to be associated with multiple CLCuD-begomoviruses in Pakistan [15,24]. These observations suggest that different recombination events in the betasatellite might be associated with CLCuD outbreaks at different time.

### Different species of alphasatellites associated with CLCuD-begomovirus complex in Northwest India

CLCuD-begomovirus associated alphasatellite molecules were amplified cloned, sequenced and analysed in the present study. They were typical alphasatellites with genome length of 1366 to 1374 nts. All the present alphasatellite molecules had three conserved features including (i) a stem-loop structure with nanonucleotides (Table 1), (ii) a Rep gene of 942 to 948 nts in the virion sense strand, encoding a putative protein of ~ 36.6 kDa with 315 aa lengths, and (iii) an adenine rich (A-rich) region of 122–187 nts length. The analysis of nucleotides and deduced amino acid sequence of the Rep gene of the present alphasatellites showed 83–100 PNI and 74–99% aa identity among themselves. In phylogenetic tree based on, the Rep gene sequences of the present alphasatellites fell into three alphasatellites GDarSLA, CLCuMA and CLCuBuA.

All the present alphasatellites contain predicted hairpin structures with a loop containing conserved nanonucleotide sequences, TAGTATTAC (positioned at 1368 to 2 nt), The stem-loop structure has a loop of 11 nts length, including the nanonucleotides (positioned at 1366 to 2 nt), and a nine base-paired stem at nt position from 1357 to 1365 with the reverse complementary position of 3 to 11 nts (Fig 6). The A-rich region of all the present alphasatellites has a typical length of 122 to 187 nts having adenine (A) content between 48.9–60.6%, compared to overall A content between 29 and 33 per cent in the whole alphasatellite genome (S4 Table).

Of the 14 isolates of alphasatellite sequence Hmg-16-1A and SG-15-11A had 100% nts sequence identity. Thus, SG-15-11A was considered for further analysis. Sequence analysis of complete genome of the present 13 alphasatellites other alphasatellites associated with CLCuD-begomoviruses and other begomoviruses. In the pair-wise sequence analysis, showed that the present alphasatellites had 71–99 PNI nt identity among themselves (Table 5). Alphasatellites, SG-14-23A, SG-15-11A, SG-16-5A, Hmg-15-6A, and Fz-15-10A shared 98–99 PNI nt identities among themselves and related with GDarSLA by 87–97 PNI identity; alphasatellites ARSB-15-1A, ARSB-15-7A, ARSF-15-1A, ARSF-15-7A and Hmg-14-1A showed 94–99 PNI among themselves relating with CLCuMA by 91–96 PNI. Alphasatellites, Fz-15-1A, Fz-15-7A and Fz-16-7A, shared 95–98 PNI among themselves and related with CLCuBuA by 93–98 PNI. Based on the demarcation cut-off value at ≥88 PNI among the species under the family *Alphasatellitidae* revised by Varsani et al. [47], the present alphasatellites represented three alphasatellite species, GDarSLA, CLCuMA and CLCuBuA. In phylogenetic analysis, substantiating with results of the nucleotide identity analysis, all the present alphasatellites were distributed under these three different alphasatellite species (Fig 5).

**Table 5:** Percent nucleotide identity matrix of complete genome of alphasatellites associated with the present and other CLCuD-begomoviruses and other host infecting begomoviruses.

Isolate	Alphasatellites of the present CLCuD-begomoviruses													Recognized alphasatellite species			Others host infecting alphasatellite species					
	1	2	3	4	5	6	7	8	9	10	11	12	13	14	15	16	17	18	19	20	21	22
CLCuMA-[IN-ARSB-15-1A] (MF141732) (1)	100	99	99	94	78	77	82	78	99	81	81	81	82	78–81	92–93	78–79	69–73	70–79	52–65	51	41	41
CLCuMA-[IN-ARSB15-7A] (MF141733) (2)		100	99	95	78	77	82	79	99	81	81	81	82	78–82	93	79	69–73	70–79	52–65	51	41	41
CLCuMA-[IN-ARSF-15-1A] (MF141734) (3)			100	95	78	77	82	78	99	81	81	81	82	78–82	92–93	78–79	69–73	70–79	52–65	51	41	41
CLCuMA-[IN-ARSF15-7A] (MF141735) (4)				100	78	77	80	79	94	80	80	80	80	77–80	91–96	79	69–72	70–81	52–65	51	41	41
CLCuBuA-[IN-Fz-15-1A] (MF141736) (5)					100	96	73	98	78	72	72	72	73	69–73	76–78	96–97	72–76	69–73	50–67	50	40	40
CLCuBuA-[IN-Fz-15-7A] (MF141737) (6)						100	75	95	77	75	75	75	75	72–75	75–78	93–94	71–76	69–72	50–67	49	40	41
GDarSLA-[IN-Fz-15-10A] (MF141738) (7)							100	71	82	98	98	98	99	88–95	81	72	66–71	69–77	53–65	52	42	41
CLCuBuA-[IN-Fz-16-7A] (MF141739) (8)								100	78	71	71	71	71	68–72	76–79	97–98	72–76	69–73	49–67	49	40	40
CLCuMA-[IN-Hmg-14-1A] (MF141740) (9)									100	81	81	81	82	78–82	92–93	78–79	69–73	70–79	52–65	51	41	41
GDarSLA-[IN-Hmg-15-6A] (MF141741) (10)										100	99	99	98	88–97	81	71–72	66–71	68–77	53–64	53	42	41
GDarSLA-[IN-SG-14-23A] (MF141742) (11)											100	99	98	88–98	80–81	71–72	66–70	68–77	53–65	53	42	41
GDarSLA-[IN-SG-15-11A] (MF141743) (12)												100	98	88–98	80–81	71–72	66–70	68–77	53–65	53	42	41
GDarSLA-[IN-SG-16-5A] (MF141744) (13)													100	87–97	81	72	66–70	68–77	53–65	53	42	41

**14: GDarSLA represents**, GDarSLA-[PK-Fai-10](FR877536), GDarSLA-[PK-KL-45-11](HE965679), GDarSLA-[PK-Mul](EU384606), CLCuVA-[IN-IARI-45Al-2-13](KM070824), GDarSLA-[IN-Rh-4-13](KM103525); **15: CLCuMA,** CLCuMA-[PK-Sha](AM711116),CLCuMA-[IN-Pun](KY783480); **16: CLCuBuA**, CLCuBuA-[PK-KL-82-11](HE972282), CLCuBuA-[PK-KL-70-11](HE972277), CLCuBuA-[IN-Raj-07](FN658728), CLCuBuA-[IN-Pun-07](FN658727), CLCuBuA-[IN-Pun-07](NC013803); **Others 14**: TYLCCNA-[CH-Yun-14](KX759649), TbCSA-[Ch-Yun-Y35](AJ579345); **15**: ChLCuA-[PK-Pun](FM179614) and OLCuA-[In-Pun-C31-15](MF929022); **16**: ToLCCMA(FR717142), CrYVMA-[IN-His-Uf-1-13](KM103524); **17**: CrYVMoA-[IN-Har-07](FN658709), **18**: AYVSGA-[SG-pGEM-AYVV2] (NC003414); **19**: EuMA- [BR-Cha18-09 (KX348227).

Earlier many alphasatellite species, like GDarSLA, CLCuBuA, CLCuMA, CrYVMoA (KC577541), CLCuLucA (HQ343234), OLCuA, ToLCA, AYVIA and GLCuA have been reported from Northwest India [14–16]. However, the classification given by Briddon et al. did not include the alphasatettites CrYVMoA, OLCuA and GLCuA in the family *Alphasatellitidae* [18]. Earlier two alphasatellite species GDarSLA and CrYVMoA are reported to be associated with CLCuD-begomovirus complex in Northwest India [6]. In the present study, three alphasatellite species, GDarSLA, CLCuMA and CLCuBuA are reported to be associated with CLCuD-begomovirus complex in Northwest India, indicating GDarSLA and CLCuBuA are prevalent in this region since their first report from Northwest India [41].

**Fig 7:**
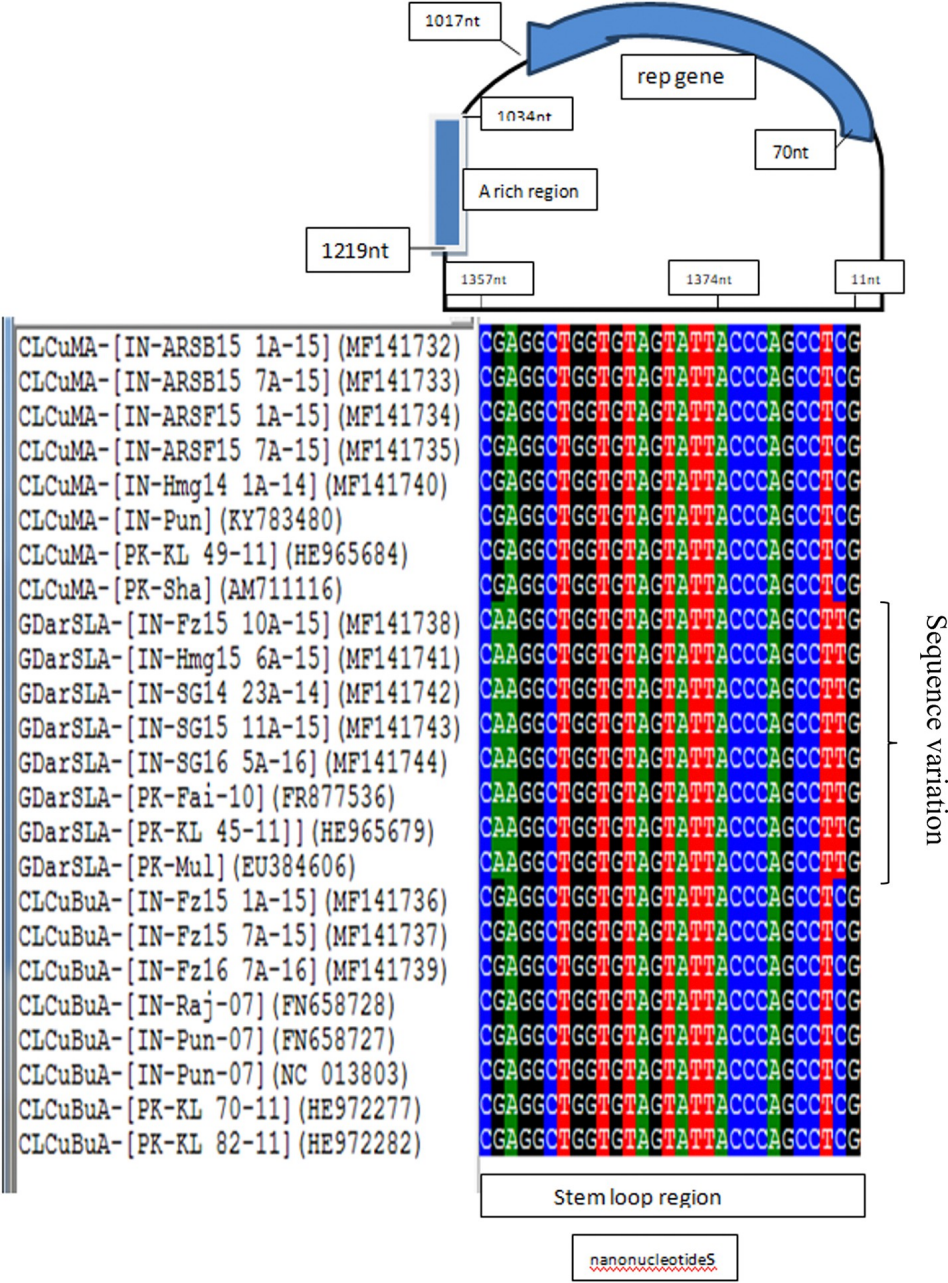
Analysis of stem-loop region of the present CLCuD-begomovirus associated alphasatellite molecules and other CLCuD-begomovirus alphasatellite molecules. The sequence region indicated in the box represent the nanonucleotide sequence.

Sequence variation in the stem-loop regions of alphasatellite species was studied. The sequence variations at two locations, one at the 1358^th^ nt and another at the 10^th^ nt position of the stem structure in the stem loop of the alphastellites were observed (Fig 7). The members of alphasatellite species CLCuBuA and CLCuMA showed G, whereas the members of alphasatellite species GDarSLA showed A at 1358^th^ nt position. The members of CLCuBuA and CLCuMA showed C, whereas GDarSLA showed T at the 10^th^ nt position which is the reverse complementary position of 1358^th^ nt. This variation could be very important to differentiate the members of the GDarSLA from members of CLCuBuA and CLCuMA.

### Inter-species recombination in alphasatellite species associated with CLCuD-begomovirus complex in Northwest India

**Fig 8:**
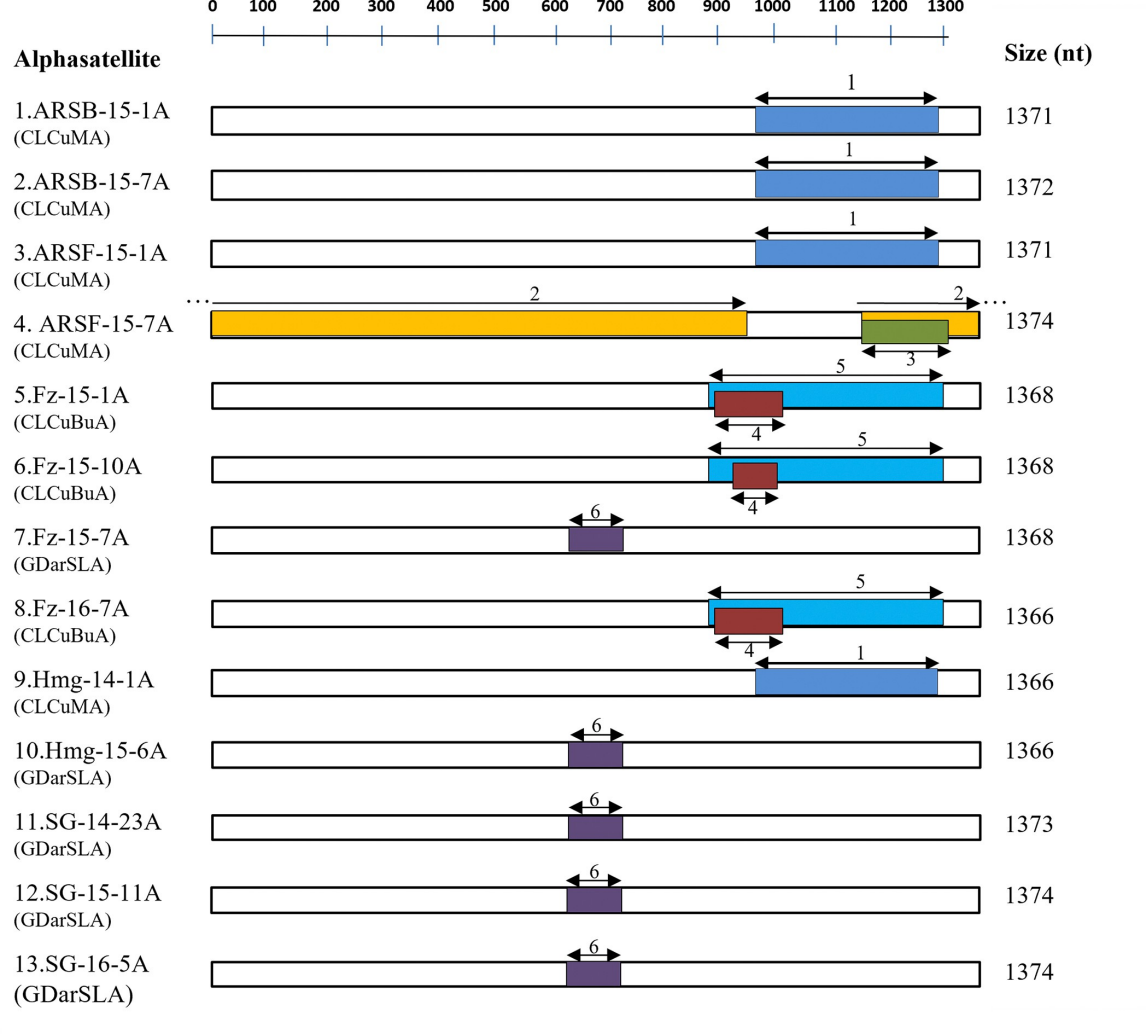
Recombination events identified in the present alphasatellites associated with CLCuD-begomovirus. Genetic map of DNA of alphasatellites molecule is shown at the top of the figure, recombinant fragments are represented by double arrow color bars along with minor parent involved in each recombination event represented by the bold number. Recombinant event with minor donor 1: CYVMA-[DarSLA-Hi-3](KM103523); 2: CLCuMA-[IN-Pun](KY783480); 3: CLCuA-[PK-KL68-11](HE966420); 4: TYLCCNA-[CH-Yun](AJ579357); 5: CLCuA-[PK-Nob-3](AJ512954); 6: TolCUPKA-[PK-KL-24-11](HT966419). Shaded areas indicating the recombinant region in genome of satellite molecule.

The RDP4 detected all the present alphasatellites as recombinants involving overall six recombination events. The Rep gene of all the present alphasatellites were found to be recombinants, albeit the patterns of recombination events in Rep gene varied (Table 6 and Fig 8). The recombination patterns in the present alphasatellites, GDarSLA, CLCuMA and CLCuBuA were different from each other. In CLCuMA, a region of 318 nts length of Rep gene from position of 974 to 1291 nt; in CLCuBuA, two regions of 423 nts and 81–142 nts length of Rep gene from position of 879–1301 nt and 897–1020 nt, respectively; and in GDarSLA, small region of 91nts length of Rep gene from position of 639–729 nt were recombinant (Table 6) indicating that recombination in the present alphasatellite GDarSLA is weak compared to other alphasatellites. Most of the present alphasatellites showed inter species recombination, where TbCSA-[CH-Yun-Y35] (AJ579345), CLCuMA and AyVSGA (NC003414) were detected as major parents for recombination of both the present alphasatellites CLCuMA and CLCuBuA (KM103526), where as CLCuBuA (KM103526) was found to be the major parent for the present alphasatellite GDarSLA (Table 6). Exceptionally, one present alphasatellite variant CLCuMA-[ARSF-15-7A] showed a large region (1305 nt length) as recombinant from position of 1141 to 945 nt and 1141 to 1305 nt (Table 6) encompassing Rep gene, A-rich and stem-loop region. The CLCuMA-[PK-Sha] (AM711116) was involved as a major parent (Table 6 and Fig 8) for this alphasatellite indicating that CLCuMA-[ARSF-15-7A] has been evolved with a strong recombination.

**Table 6:** Recombination analysis of alphasatellite molecule associated with CLCuD-begomovirus using the RDP 4.6.

Alphasatellite (Species)	Recombination site	Major parent x Minor parent	Length (nt)	Event number	Detected by method*	P Value (Max)**
1.ARSB-15-1A (CLCuMA)	974–1291 (Part of Rep)	TbCSA-[CH-Yun-Y35] (AJ579345) x CYVMA-[IN-Hi-3] (KM103523)	318	1	R, G, B, M, C, S, 3S, L	1.5 x10^-3^
2.ARSB-15-7A (CLCuMA)	974–1291 (Part of Rep)	TbCSA-[CH-Yun-Y35] (AJ579345) x CYVMA-[IN-Hi-3] (KM103523)	318	1	R, G, B, M, C, S, 3S, L	1.5 x10^-3^
3.ARSF-15-1A (CLCuMA)	974–1291 (Part of Rep)	TbCSA-[CH-Yun-Y35] (AJ579345) x CYVMA-[IN-Hi-3] (KM103523)	318	1	R, G, B, M, C, S, 3S, L	1.5 x10^-3^
4.ARSF-15-7A (CLCuMA)	1141–945 (Comlete Rep)	CLCuMA-[PK-Sha] (AM711116) x CLCuMA-(IN-Pun) (KY783480)	1149	2	R, G, B, M, C, S, 3S, L	4.0 x10^-5^
	1141–1305 (Part of Rep)	CLCuMA-(IN-Pun) (KY783480) x OLCuA-[PK-KL68-11] (HE966420)	165	3	R, G, B, M, C, S, 3S, L	2.2 x10^-5^
5.Fz-15-1A (CLCuBuA)	897–1020 (Part of Rep)	AYVSGA-[SG-pGEM-AYVV2] (NC003414) x TYLCCNA-[CH-Yun] (AJ579357)	142	4	R, G, B, M, C, S, 3S, L	1.3 x10^-2^
	879–1301 (Part of Rep)	CLCuMA-[IN-ARSF15-7] (MF141735) x (OLCuA-[PK-Nob-3] (AJ512954)	423	5	R, G, B, M, C, S, 3S, L	1.5 x10^-3^
6.Fz-15-7A (CLCuBuA)	925–1005 (Part of Rep)	AYVSGA-[SG-pGEM-AYVV2] (NC003414) x TYLCCNA-[CH-Yun] (AJ579357)	81	4	R, G, B, M, C, S, 3S, L	1.3 x10^-2^
	879–1301 (Part of Rep)	CLCuMA-[IN-ARSF15-7] (MF141735) x OLCuA-[PK-Nob-3] (AJ512954)	423	5	R, G, B, M, C, S, 3S, L	1.5 x10^-3^
7.Fz-15-10A (GDarSLA)	639–729 (Part of Rep)	CLCuBuA -[IN-Sa-3] (KM103526) x TolCUPKA-(PK-KL-24-11) (HT966419)	91	6	R, G, B, 3S	2.8 x10^-4^
8.Fz-16-7A (CLCuBuA)	897–1020 (Part of Rep)	AYVSGA-[SG-pGEM-AYVV2] (NC003414) x TYLCCNA-(CH-Yun) (AJ579357)	142	4	R, G, B, M, C, S, 3S, L	1.3 x10^-2^
	879–1301 (Part of Rep)	CLCuMA-[IN-ARSF15-7] (MF141735) x OLCuA-[PK-Nob-3] (AJ512954)	423	5	R, G, B, M, C, S, 3S, L	1.5 x10^-3^
9.Hmg-14-1A (CLCuMA)	974–1291 (Part of Rep)	TbCSA-(CH-Yun-Y35) (AJ579345) x CYVMA-[IN-Hi-3] (KM103523)	318	1	R, G, B, M, C, S, 3S, L	1.5 x10^-3^
10.Hmg-15-6A (GDarSLA)	639–729 (Part of Rep)	CLCuBuA -[IN-Sa-3] (KM103526) x TolCuPKA-(PK-KL-24-11) (HE966419)	91	6	R, G, B, 3S	2.8 x10^-4^
11.SG-14-23A (GDarSLA)	639–729 (Part of Rep)	CLCuBuA -[IN-Sa-3] (KM103526)) x TolCuPKA-(PK-KL-24-11) (HE966419)	91	6	R, G, B, 3S	2.8 x10^-4^
12.SG-15-11A (GDarSLA)	639–729 (Part of Rep)	CLCuBuA -[IN-Sa-3] (KM103526) x TolCuPKA-(PK-KL-24-11) (HE966419)	91	6	R, G, B, 3S	2.8 x10^-4^
13.SG-16-5A (GDarSLA)	639–729 (Part of Rep)	CLCuBuA -[IN-Sa-3] (KM103526) x TolCuPKA-(PK-KL-24-11) (HE966419)	91	6	R, G, B, 3S	2.8 x10^-4^

*: B: Bootscan, C: Chimera, G: Geneconv,L: LARD, M: Maxchi, R: RDP, Si: Siscan and 3-S: 3SEQ implemented in the RDP4**: Highest acceptable P-value cut-off of 0.05 detected the evidences of recombination events among the sequences.

## Conclusions

Begomoviruses are the largest group of plant viruses which involve rapid recombination and pseudo-recombination to breakdown the host resistance and develop disease symptoms. CLCuD-begomovirus associated with betasatellite and alphasatellie molecules is major constraint in productivity of cotton in Northwest India, although degree of disease incidence has been changing year after year. In the present study a single and genetically divergent betasatelite species CLCuMB, and three divergent alphasatellite species, CLCuMA, CLCuBuA and GDarSLA, are identified. Intra species recombination in betasatellites and inter species recombination in alphasatellites are the major phenomenon for evolution of new satellite molecules in CLCuD-begomovirus complex in Northwest India. The study underlines the significance of the Indian subcontinent as a hot belt for genetic variabilities of begomoviruses and its satellite molecules leading to develop a new species or variant. Therefore, it is concluded that the recombinant betasatellite and alphasatellite in association with CLCuD-begomoviruses are the important causes for changing the disease severity and outbreak of CLCuD in Northwest India.

## Supporting information

S1 Table Cotton leaf curl disease incidence and per cent disease index in Northwest India during 2014–2016. (DOCX)

S2 Table Satellite conserved region of the present betasatellites. (DOCX)

S3 Table Analysis of potential adenine (A)- rich region of present betasatellites. (DOCX)

S4 Table Analysis of potential adenine(A)-rich region of present alphasatellites. (DOCX)

## References

[1] Anonymous. Ministry of Textiles, Govt. of India.2021. https://www.texmin.nic.in.

[2] Briddon R.W., Mansoor S., Bedford I.D., Pinner M.S., Saunders K., Stanley J., et al. Identification of DNA components required for induction of cotton leaf curl disease. Virology. 2001. 285: 234–243. doi: 10.1006/viro.2001.0949 . 11437658

[3] Rajagopalan P.A., Naik A., Katturi P., Kurulekar M., Kankanallu R.S., and Anandalakshmi R. Dominance of resistance-breaking cotton leaf curl Burewala virus (CLCuBuV) in Northwestern India. Archives of Virology. 2002. 157, 855–868. doi: 10.1007/s00705-0122-12225-y 22307170

[4] Sattar M.N., Kvarnheden A., Saeed M., and Briddon R.W. Cotton leaf curl disease-an emerging threat to cotton production worldwide. Journal of General Virology.2013. 94, 695–710. doi: 10.1099/vir.0.049627-0 23324471

[5] Sattar M.N., Iqbal Z., Tahir M.N., and Ullah S. The Prediction of a New CLCuD epidemic in the Old World. Frontiers in Microbiology. 2017. 8: 631. doi: 10.3389/fmicb.2017.00631 28469604 PMC5395620

[6] Biswas K.K., Bhattacharyya U.K., Palchoudhury S., Balram N., Kumar A., Arora R., et al. Dominance of recombinant cotton leaf curl multan-Rajasthan virus associated with cotton leaf curl disease outbreak in Northwest India. PLoS ONE. 2020. 15(4):e0231886. doi: 10.1371/journal.pone.0231886 32320461 PMC7176085

[7] Monga D., Manocha V., Chandkumar K., Seni K., and Singh N.P. Occurrence and prediction of cotton leaf curl virus disease in northern zone. Journal Cotton Research. 2011. 25: 273–277.

[8] Bhattacharyya U.K., Godara S., Kumar P., Monga D., and Biswas K.K. Recent status and distribution pattern of cotton leaf curl disease in Northwest India: an alarming situation in future cotton cultivation. Indian Journal of Agricultural Science. 2017. 87(5):624–633.

[9] Balram N., Bhattacharyya U.K., Aman S., Pradeep K., Sandeep R., Rupesh A., et. al., Current distribution and severity of leaf curl begomovirus disease in cotton growing areas of Northwest India. Journal of Mycology and Plant Pathology. 2017. 47:7 259–272.

[10] Godara S, Saini N., Khurana S.M.P., and Biswas K.K. Lack of resistance in cotton against cotton leaf curl begomovirus disease complex and occurrence of natural virus sequence variants. Indian Phytopathology. 2015. 68: 326–333.

[11] Varma A., Malathi V.G., Handa A., Aiton M., Harrison B.D., Verma J.P., et al. Occurrence of leaf-curl of cotton and okra in Northern India. In: Abstract of the 6th International Congress of Plant Pathology, Montreal, 1993. 17.5.14.

[12] Ajmera B.D. Occurrence of leaf curl virus on American cotton (*G. hirsutum*) in North Rajasthan. Paper printed in National Seminar Cotton production challenges in 21stCentury at C.C.S.H.A.U., Hissar: abstract. 1994. Page 86.

[13] Brown J.K., Zia Ur-Rehman M., Avelara S., Chingandu N., Hameed U., Haider S., et al. Molecular diagnostic development for begomovirus-betasatellite complexes undergoing diversification: A case study. Virus Research. 2017. 241: 29–41. doi: 10.1016/j.virusres.2017.04.014 . 28438632

[14] Godara S., Paul Khurana S.M., and Biswas K.K. Three variants of cotton leaf curl begomoviruses with their satellite molecules are associated with cotton leaf curl disease aggravation in New Delhi. Journal of Plant Biochemistry and Biotechnology. 2016. 26, 97–105. doi: 10.1007/s135622-016-0370-x

[15] Datta S., Budhauliya R., Das B., Gopalakrishnan R., Sharma S., Chatterjee S., et al. Rebound of Cotton leaf curl Multan virus and its exclusive detection in cotton leaf curl disease outbreak, Punjab (India). Scientific Report. 2017. 7: 17361. doi: 10.1038/s41598-017-17680-9 . 29234082 PMC5727119

[16] Qadir R., Khan Z.A., Monga D., and Khan J.A. Diversity, and recombination analysis of Cotton leaf curl Multan virus: a highly emerging begomovirus in northern India. BMC genomics. 2019. 20: 274. doi: 10.1186/s12864-019-5640-2 . 30954067 PMC6451280

[17] Zubair M., Zaidi S.S., Shakir S., Farooq M., Amin I., Scheffler J.A., et al., Multiple begomoviruses found associated with cotton leaf curl disease in Pakistan in early 1990 are back in cultivated cotton. Scientific Reports. 2017a. 7: 680 doi: 10.1038/s41598-017-00727-2 28386113 PMC5429635

[18] Briddon R.W., and Stanley J. Sub-viral agents associated with plant single-stranded DNA viruses. Virology. 2006. 344, 198–210. doi: 10.1016/j.virol.2005.09.042 16364750

[19] Saeed M., Zafar Y., Randles J.W., and Rezaian M.A. A monopartite begomovirus-associated DNA b satellite substitutes for the DNA B of bipartite begomovirus to permit systemic infection. Journal of General Virology. 2007. 88, 2881–2889. doi: 10.1099/vir.0.83049-0 17872543

[20] Amin I., Hussain K., Akbergenov R., Yadav J.S., Qazi J., Mansoor S., et al. Suppressors of RNA silencing encoded by the components of the cotton leaf curl begomovirus-betasatellite complex. Molecular Plant Microbe Interaction. 2011. 24: 973–983. doi: 10.1094/MPMI-01-11-0001 21751853

[21] Briddon R. W., Bull S.E., Amin I., Idris A.M., Mansoor S., Bedford I.D., et al. Diversity of DNA 1: a satellite-like molecule associated with monopartite begomovirus-DNA β complexes. Virology. 2004. 324: 462–474. doi: 10.1016/j.virol.2004.03.041 . 15207631

[22] Mansoor S., Amrao L., Amin I., Briddon R.W., Malik K.A., and Zafar Y. First report of cotton leaf curl disease in central and southern Sindh province in Pakistan. Plant Disease. 2006. 90: 826. doi: 10.1094/PD-90-0826A . 30781247

[23] Siddiqui K., Mansoor S., Briddon R.W., and Amin I. Diversity of alphasatellites associatedwith cotton leafcurl disease in Pakistan. Virology Reports. 2016. 6: 41–52. doi: 10.1016/j.virep.2016.05.004

[24] Zubair M., Zaidi S.S., Shakir S., Amin I., and Mansoor S. Insights into Cotton leaf curl Multan betasatellite, the most important component of cotton leaf curl disease complex. Viruses. 2017. 9, 280. 28961220 10.3390/v9100280 PMC5691632

[25] Saleem H., Nahid N., Shakir S., Ijaz S., Murtaza G., Khan A.A., et al. Diversity, mutation and recombination analysis of cotton leaf curl geminiviruses. PLoS ONE. 2016. 11:e0151161. doi: 10.1371/journal.pone.0151161 26963635 PMC4872795

[26] Briddon R.W., Martin D.P., Roumagnac P., Navas-Castillo J., Fiallo-Olive E., Moriones E., et al. *Alphasatellitidae*: a new family with two subfamilies for the classification of geminivirus- and nanovirus-associated alphasatellites. Archives of Virology. 2018. 163: 2587–2600. doi: 10.1007/s00705-018-3854-2 . 29740680

[27] Anonymous. ICAR-AICRP (cotton) Annual Report (2015–16). ICAR–All India Coordinated Research Project (cotton), Coimbatore-641003, Tamil Nadu. 2016. https://aiccip.cicr.org.in/mainaiccip_reports.html.

[28] Doyle J.J. and Doyle J.L. Isolation of plant DNA from fresh tissue. Focus. 1990. 12(1): 13–15.

[29] Haible D., Kober S. and Jeske H. Rolling circle amplification revolutionizes diagnosis and genomics of Geminiviruses. Journal of Virological Methods. 2006. 135: 9–16. doi: 10.1016/j.jviromet.2006.01.017 . 16513183

[30] Briddon R.W., Bull S.E., Mansoor S., Amin I., and Markham P.G. Universal primers for the PCR mediated amplification of DNA beta; a molecule associated with some monopartite begomovirus. Molecular Biotechnology. 2002. 20, 315–318. doi: 10.1385/MB:20:3:315 . 11936260

[31] Bull S.E., Briddon R.W., and Markham P.G. Universal primers for the PCR-mediated amplification of DNA 1: a satellite-like molecule associated with begomovirus-DNA β complexes. Molecular Biotechnology. 2003. 23, 83–86. doi: 10.1385/MB:23:1:83 . 12611272

[32] Biswas K.K., Tarafdar A., Diwedi S., and Lee R.F. Distribution, genetic diversity and recombination analysis of *Citrus tristeza virus* of India. Virus Gene. 2012. 45: 139–148. 10.1007/s11262-012-0748-3 22562224

[33] Hall T.A. BioEdit: A user-friendly biological sequence alignment editor and analysis program for Windows 95/98/NT. Nucleic Acids Symposium. Series. 1999; 41: 95–98.

[34] Thompson J.D., Gibson T.J., Plewniak F., Jeanmougin F., and Higgins D.G. The Clustal X windows interface; flexible strategies for multiple sequence alignment aided by quality analysis tools. Nucleic Acids Research. 1997. 25: 4876–4882. doi: 10.1093/nar/25.24.4876 . 9396791 PMC147148

[35] Tamura K., Stecher G., and Kumar S. MEGA 11: Molecular Evolutionary Genetics Analysis Version 11. Molecular Biology Evolution. 2021. 38(7): 3022–3027. doi: 10.1093/molbev/msab120 PMC8233496 33892491

[36] Saitou N. and Nei M. The neighbour-joining method: A new method for reconstructing phylogenetic trees. Molecular Biology Evolution. 1987. 4:406–425. doi: 10.1093/oxfordjournals.molbev.a040454 3447015

[37] Muhire B.M., Varsani A., and Martin D.P. SDT: A virus classification tool based on pairwise sequence alignment and identity calculation. PLoS ONE. 2014. 9. e108277 doi: 10.1371/journal.pone.0108277 . 25259891 PMC4178126

[38] Martin D.P., Murrell B., Golden M., Khoosa A., and Muhire B. RDP4: Detection and analysis of recombination patterns in virus genomes. Virus Evolution. 2015. 1(1): vev003. doi: 10.1093/ve/vev003 27774277 PMC5014473

[39] Adams M.J., Lefkowitz E.J., King A.M.Q., Harrach B., Harrison R.L., Knowles N.J., et al. Changes to taxonomy and the international code of virus classification and nomenclature ratified by the international committee on taxonomy of viruses. Archives of Virology. 2018. 163 (9):2601–2631. doi: 10.1007/s00705-018-3847-1 29754305

[40] Briddon R.W., Brown J.K., Moriones E., Stanley J., Zerbini M., Zhou X. et al. Recommendations for the classification and nomenclature of the DNA-b satellites of begomoviruses. Archives of Virology. 2008. 153:763–781. doi: 10.1007/s00705-007-0013-6 18247103

[41] Zaffalon V., Mukherjee S.K., Reddy V.S., Thompson J.R., and Tepfer M. A survey of *Geminiviruses* and associated satellite DNAs in the cotton-growing areas of Northwestern India. Archives of Virology. 2012. 157: 483–495. doi: 10.1007/s00705-011-1201-y . 22209785

[42] Briddon R.W., Bull S.E., Amin I., Idris A.M., Mansoor S., Bedford I.D., et al. Diversity of DNA beta, a satellite molecule associated with some monopartite begomoviruses. Virology. 2003. 312: 106–121. doi: 10.1016/s0042-6822(03)00200-9 . 12890625

[43] Mansoor S., Amin I., Iram S., Hussain M., Zafar Y., Malik K. A., et al. Breakdown of resistance in cotton-to-cotton leaf curl disease in Pakistan. Plant Pathology. 2003. 52: 784 doi: 10.1111/j.1365-3059.2003.00893.x

[44] Amin I., Mansoor S., Amrao L., Hussain M., Irum S., Zafar Y, et al. Mobilisation into cotton and spread of a recombinant cotton leaf curl disease satellite. Archives of Virology. 2006. 151, 2055–2065. doi: 10.1007/s00705-006-0773-4 16732497

[45] Amrao L., Amin I., Shahid S.M., Briddon R.W., and Mansoor S. Cotton leaf curl disease in resistant cotton is associated with a single begomovirus that lacks an intact transcriptional activator protein. Virus Research. 2010b. 152, 153–163. do: doi: 10.1016/j.virusres.2010.06.019 20600387

[46] Amrao L., Akhter S., Tahir M.N., Amin I., Briddon R.W., and Mansoor S. Cotton leaf curl disease in Singh province of Pakistan is associated with recombinant begomovirus components. Virus Research. 2010a. 153, 161–165. doi: 10.1007/s00705-010-0760-7 20621137

[47] Varsani A., Martin D.P., Randles J.W. et al. Taxonomy update for the family *Alphasatellitidae*: new subfamily, genera, and species. *Arch Virol* 166, 3503–3511 (2021). 10.1007/s00705-021-05232-6. 34550466

