# Supplementary material for: Divergent Cotton leaf curl Multan betasatellite and three different alphasatellite species associated with cotton leaf curl disease outbreak in Northwest India
Source: PLoS One. 2025 Jan 9;20(1):e0313844. doi: 10.1371/journal.pone.0313844 (PMC11717315; doi:10.1371/journal.pone.0313844)
Supplement: S1 Table — (DOCX) [file pone.0313844.s001.docx]

**S1Table.** Cotton leaf curl disease incidence and per cent disease index in Northwest India during 2014-2016.

| **District** | **Geographical coordinates** | **2014** | | | **2015** | | | **2016** | | |
| --- | --- | --- | --- | --- | --- | --- | --- | --- | --- | --- |
|  | **Latitude/ Longitude** | **No. of field surveyed** | **Disease***  **Incidence (%)** | **PDI (%)** | **No. of field surveyed** | **Disease***  **Incidence (%)** | **PDI (%)** | **No. of field surveyed** | **Disease***  **Incidence (%)** | **PDI (%)** |
| **Rajasthan** | | | | | | | | | | |
| Sri Ganganagar | 29° 49' N/ 73° 50' E | 21 | 4.6 | 3.4 | 19 | 55.2 | 27.9 | 26 | 65.2 | 30.1 |
| Hanumangarh | 29° 35' N/ 74° 21' E | 19 | 7.0 | 2.4 | 13 | 32.6 | 12.5 | 17 | 23.0 | 8.6 |
| **Punjab** | | | | | | | | | | |
| Fazilka | 30° 25' N/ 74° 04' E | 28 | 45.7 | 12.9 | 66 | 52.9 | 36.2 | 54 | 44.6 | 23.8 |
| Bhatinda | 30° 20' N/ 74° 95' E | 15 | 48.0 | 11.7 | 24 | 62.9 | 36.9 | 15 | 51.6 | 23.2 |
| Faridkot | 30° 40' N/ 74° 45' E | 12 | 97.1 | 27.5 | 28 | 72.3 | 46.8 | 27 | 70.4 | 46.3 |

* Average incidence of CLCuD calculated based on observation of 100-200 CLCuD infected plants/ field
