# Supplementary material for: Divergent Cotton leaf curl Multan betasatellite and three different alphasatellite species associated with cotton leaf curl disease outbreak in Northwest India
Source: PLoS One. 2025 Jan 9;20(1):e0313844. doi: 10.1371/journal.pone.0313844 (PMC11717315; doi:10.1371/journal.pone.0313844)
Supplement: S2 Table — (DOCX) [file pone.0313844.s002.docx]

**S2 Table. Satellite conserved region of the present betasatellites**

| **Isolate** | **Position** | **Length** | **SCR sequence** |
| --- | --- | --- | --- |
| ARSB-15-1B (KY523512) | 1246-17 | 129 | CCGGCATCAATTTACGACACGCGCGGCGGTGTGTACCCCTGGGAGGGTAGTAATATACGCTACGCAGCAGCCTTAGCTACGCCGGAGCTTAGCTCGCCCACGCTTTAATATTACCGTGGGCGAGCGGTG |
| ARSB-15-7B (KY523513) | 1244-17 | 130 | CCGGCATCAATTTACGACACGCGCGGCGGTGTGTACCCCTGGGAGGGTAGTAATACTACGCTACGCAGCAGCCTTAGCTACGCCGGAGCTTAGCTCGCCCACGCTTTAATATTACCGTGGGCGAGCGGTG |
| ARSF-15-1B (KY523514) | 1245-17 | 130 | CCGGCATCAATTTACGACACGCGCGGCGGTGTGTACCCCTGGGAGGGTAGTAATACTACGCTACGCAGCAGCCTTAGCTACGCCGGAGCTTAGCTCGCCCACGCTTTAATATTACCGTGGGCGAGCGGTG |
| ARSF-15-7B (KY523515) | 1245-17 | 130 | CCGGCATCAATTTACGACACGCGCGGCGGTGTGTACCCCTGGGAGGGTAGTAATACTACGCTACGCAGCAGCCTTAGCTACGCCGGAGCTTAGCTCGCCCACGCTTTAATATTACCGTGGGCGAGCGGTG |
| Fz-15-1B (KY523516) | 1243-17 | 131 | CCGGCATCAATTTACGACACGCGCGGCGGTGTGTACCCCTGGGAGGGTAGGTACCACTACGCTACGCAGCAGCCTTAGCTACGCCGGAGCTTAGCTCGCCCACGCTTTAATATTACCGTGGGCGAGCGGTG |
| Fz-15-10B (KY523517) | 1243-17 | 131 | CCGGCATCAATTTACGACACGCGCGGCGGTGTGTACCCCTGGGAGGGTAGGTACCACTACGCTACGCAGCAGCCTTAGCTACGCCGGAGCTTAGCTCGCCCACGCTTTAATATTACCGTGGGCGAGCGGTG |
| Hmg-14-1B(KY523518) | 1244-17 | 131 | CCGGCATCAATTTACGACACGCGCGGCGGTGTGTACCCCTGGGAGGGTAGGTACCACTACGCTACGCAGCAGCCTTAGCTACGCCGGAGCTTAGCTCGCCCACGCTTTAATATTACCGTGGGCGAGCGGTG |
| Hmg-15-6B(KY523519) | 1244-17 | 131 | CCGGCATCAATTTACGACACGCGCGGCGGTGTGTACCCCTGGGAGGGTAGACACCACTACGCTACGCAGCAGCCTTAGCTACGCCGGAGCTTAGCTCGCCCACGCTTTAATATTACCGTGGGCGAGCGGTG |
| Hmg-16-1B(MF141731) | 1244-17 | 130 | CCGGCATCAATTTACCACAACCCGCGGCGTGTGAACCCCTGGGAGGGTAGAAACCCTACGCTACGCAGCAGCCTTAGCTACGCCGGAGCTTAGCTCGCCCACGCTTTAATATTACCGTGGGCGAGCGGTG |
| SG-14-23B(KY523520) | 1245-17 | 131 | CCGGCATCAATTTACGACACGCGCGGCGGTGTGTACCCCTGGGAGGGTAGGTACCACTACGCTACGCAGCAGCCTTAGCTACGCCGGAGCTTAGCTCGCCCACGCTTTAATATTACCGTGGGCGAGCGGTG |
| SG-15-11B(KY523521) | 1244-17 | 131 | CCGGCATCAATTTACGACACGCGCGGCGGTGTGTACCCCTGGGAGGGTAGGTACCACTACGCTACGCAGCAGCCTTAGCTACGCCGGAGCTTAGCTCGCCCACGCTTTAATATTACCGTGGGCGAGCGGTG |
| SG-16-1B(MF141730) | 1243-17 | 129 | CCGGCATCAATTACGAACGCGCGGCGGTGTGTACCCCTGGGAGGGTAGGTACCACTACGCTACGCAGCAGCCTTAGCTACGCCGGAGCTTAGCTCGCCCACGCTTTAATATTACCGTGGGCGAGCGGTG |
