# Supplementary material for: Divergent Cotton leaf curl Multan betasatellite and three different alphasatellite species associated with cotton leaf curl disease outbreak in Northwest India
Source: PLoS One. 2025 Jan 9;20(1):e0313844. doi: 10.1371/journal.pone.0313844 (PMC11717315; doi:10.1371/journal.pone.0313844)
Supplement: S3 Table — (DOCX) [file pone.0313844.s003.docx]

**S3 Table. Analysis of potential adenine (A)- rich region of present betasatellites**

| **Isolate** | **Position** | **Length (nt)** | **No. of A** | **A-Rich regions in the sequences** | **% of A-content** |
| --- | --- | --- | --- | --- | --- |
| ARSB-15-1B  (KY523512) | 768-981 | 214 | 130 | AAAAGGAAAAACGGAACTGAAGAGGAAAAACAAAGAAAAGAAACAAGGATATATTATTTATGAAAGAAATGGGAGCGCAGCGAATCAAAACAGGAAAACCCAAGGAAAGAGAAAAAATAAAAAGGAAAGAGAAAAAATAAAAAGTAAAGAGAAAAAAAATATAAATTCGAAAACGTCATCGTTTGAGAAGAGGGAAAAAAAAGAAAAAACAAAA | 60.7 |
| ARSB-15-7B  (KY523513) | 766-979 | 214 | 130 | AAAAGGAAAAACGGAACTGAAGAGGAAAAACAAAGAAAAGAAACAAGGATATATTATTTATGAAAGAAATGGGAGCGCAGCGAATCAAAACAGGAAAACCCAAGGAAAGAGAAAAAATAAAAAGGAAAGAGAAAAAATAAAAAGTAAAGAGAAAAAAAATATAAATTCGAAAACGTCATCGTTCGAGAAGAGGGAAAAAAAAGAAAAAACAAAA | 60.7 |
| ARSF-15-1B  (KY523514) | 767-980 | 214 | 128 | AAAAGGAAAAACGGAACTGAAGAGGAAAAACAAAGAAGCGAAACAAGGATATATTATTTATGAAAGAAATGGGAGCGCAGCGAATCAAAACAGGAAAACCCAAGGAAAGAGAAAAAATAAAAAGGAAAGAGAAAAAATAAAAAGTAAAGAGAAAAAAAATATAAATTCGAAAACGTCATCGTTTGAGAAGAGGGAAAAAAAAGAAAAAACAAAA | 59.8 |
| ARSF-15-7B  (KY523515) | 767-980 | 214 | 130 | AAAAGGAAAAACGGAACTGAAGAGGAAAAACAAAGAAAAGAAACAAGGATATATTATTTATGAAAGAAATGGGAGCGCAGCGAATCAAAACAGGAAAACCCAAGGAAAGAGAAAAAATAAAAAGGAAAGAGAAAAAATAAAAAGTAAAGAGAAAAAAAATATAAATTCGAAAACGTCATCGTTTGAGAAGAGGGAAAAAAAAGAAAAAACAAAA | 60.7 |
| Fz-15-1B  (KY523516) | 765-978 | 214 | 130 | AAAAGGAAAAACGGAACTGAAGAGGAAAAACAAAGAAAAGAAACAAGGATATATTATTTATGAAAGAAATGGGAGCGCAGCGAATCAAAACAGGAAAACCCAAGGAAAGAGAAAAAATAAAAAGGAAAGAGAAAAAATAAAAAGTAAAGAGAAAAAAAATATAAATTCGAAAACGTCATCGTTTGAGAAGAGGGAAAAAAAAGAAAAAACAAAA | 60.7 |
| Fz-15-10B  (KY523517) | 765-978 | 214 | 130 | AAAAGGAAAAACGGAACTGAAGAGGAAAAACAAAGAAAAGAAACAAGGATATATTATTTATGAAAGAAATGGGAGCGCAGCGAATCAAAACAGGAAAACCCAAGGAAAGAGAAAAAATAAAAAGGAAAGAGAAAAAATAAAAAGTAAAGAGAAAAAAAATATAAATTCGAAAACGTCATCGTTTGAGAAGAGGGAAAAAAAAGAAAAAACAAAA | 60.7 |
| Hmg-14-1B  (KY523518) | 766-979 | 214 | 127 | AAAAGGAAAAACGGAACTGAGGAAGGAAAACAAAGAAAAGAAACATGGATATATTATTTATGAAAGAAATGGGAGCGCAGCGAATCCAAACAGAAAAAGCCAAGGAAAGAGAAAAAATAAAAAGGAAAGAGAAAAAATAAAAAGTAAAGAGAAAAAAAATATAAATTCGAAAACGTCATCGTTTGAGAGGAGGGAAAAAAAAGAAAAAACAAAA | 59.3 |
| Hmg-15-6B  (KY523519) | 766-979 | 214 | 130 | AAAAGGAAAAACGGAACTGAAGAGGAAAAACAAAGAAAAGAAACAAGGATATATTATTTATGAAAGAAATGGGAGCGCAGCGAATCAAAACAGGAAAACCCAAGGAAAGAGAAAAAATAAAAAGGAAAGAGAAAAAATAAAAAGTAAAGAGAAAAAAAATATAAATTCGAAAACGTCATCGTTTGAGAGGAGGGAAAAAAAAGAAAAAACAAAA | 60.7 |
| Hmg-16-1B  (MF141730) | 766-979 | 214 | 125 | AAAAGGAAAAACGGAACTGAAGAGGAAAAACAAAGAAAAGAAACAAGGATATTTTTTTTTTGAAAGAAATGGGAGCGCAGCGAATCAAACCAGGAAAACCCAAGGAAAGAGAAAAAATAAAAAGGAAAGGGAAAAAATAAAAAGTAAAGAGAAAAAAAATATAAATTCGAAAAGGTCATCGTTTGAGAAGAGGGAAAAAAAAGAAAAAACAAAA | 58.4 |
| SG-14-23B  (KY523520) | 767-980 | 214 | 130 | AAAAGGAAAAACGGAACTGAAGAGGAAAAACAAAGAAAAGAAACAAGGATATATTATTTATGAAAGAAATGGGAGCGCAGCGAATCAAAACAGGAAAACCCAAGGAAAGAGAAAAAATAAAAAGGAAAGAGAAAAAATAAAAAGTAAAGAGAAAAAAAATATAAATTCGAAAACGTCATCGTTTGAGAAGAGGGAAAAAAAAGAAAAAACAAAA | 60.7 |
| SG-15-11B  (KY523521) | 766-979 | 214 | 130 | AAAAGGAAAAACGGAACTGAAGAGGAAAAACAAAGAAAAGAAACAAGGATATATTATTTATGAAAGAAATGGGAGCGCAGCGAATCAAAACAGGAAAACCCAAGGAAAGAGAAAAAATAAAAAGGAAAGAGAAAAAATAAAAAGTAAAGAGAAAAAAAATATAAATTCGAAAACGTCATCGTTTGAGAAGAGGGAAAAAAAAGAAAAAACAAAA | 60.7 |
| SG-16-5B  (MF141731) | 766-976 | 211 | 119 | AAAAGGAAAAACGGAACTGAGAGGACAAAGAGAAAAGAAACAAGGGTTTTTTTTTTTTGAAAGAAATGGGAGCGCAGCAAATCGAAACAGGAAACCCCAAGGAAAGAGAAAAAATAAAAAGGAAAAGGGAAAAATAAAAAGTAAAGAGAAAAAAAATATAAATTCGAAAACGTCATCGTTTGAGAAGAGGGAAAAAAAAGAAAAAACAAAA | 56.4 |
