# Supplementary material for: Divergent Cotton leaf curl Multan betasatellite and three different alphasatellite species associated with cotton leaf curl disease outbreak in Northwest India
Source: PLoS One. 2025 Jan 9;20(1):e0313844. doi: 10.1371/journal.pone.0313844 (PMC11717315; doi:10.1371/journal.pone.0313844)
Supplement: S4 Table — (DOCX) [file pone.0313844.s004.docx]

**S4 Table. Analysis of potential adenine(A)-rich region of present alphasatellites**

| **Isolate** | **Position** | **Length (nt)** | **No. of A** | **A-Rich regions in the sequences** | **% of A-content** |
| --- | --- | --- | --- | --- | --- |
| ARSB-15-1A  (MF141732) | 1034-1156 | 122 | 73 | AAAACAAGAAAGAATGAAATAAAAAAAGAAAAAGGAAAAACGTGAGCCTTCTATTAATTTAAGAAAGAAAGTGCCGCGCAGCGGCATTCAAAAAAAAATATAAATATAAAAAAAGAAAAAAA | 59.8 |
| ARSB-15-7A  (MF141733) | 1034-1157 | 122 | 74 | AAAACAAGAAAGAATGAAATAAAAAAAGAAAAAGGAAAAACGTGAGCCTTCTATTAATTTAAGAAAGAAAGTGCCGCGCAGCGGCATTCAAAAAAAAATATAAATATAAAAAAAGAAAAAAA | 60.6 |
| ARSF-15-1A  (MF141734) | 1034-1156 | 122 | 73 | AAAACAAGAAAGAATGAAATAAAAAAAGAAAAAGGAAAAACGTGAGCCTTCTATTAATTTAAGAAAGAAAGTGCCGCGCAGCGGCATTCAAAAAAAAATATAAATATAAAAAAAGAAAAAAA | 59.8 |
| ARSF-15-7A  (MF141735) | 1034-1157 | 123 | 71 | AAAACAAGAAAGAATGAAATGAAAAAAAATAAAGAAAAAACATGAGCCTTCTATTATTTCAAGATAAAACGTTGCCGCGCAGCGGCATTCAAAAAAAAAGATAAATAAAAAAAGAATATAAAA | 57.7 |
| Fz-15-1A  (MF141736) | 1043-1218 | 175 | 93 | AATAGATTATATTAAAGAGAGAAGGGCCGCGCAGCGGCAATAACATGAGTAATTTTGCTTCTTGAAAACAAGAAGGAATGAAATGAAAAAAAGAAATAAAAAAAAAAAAATGTAATATAAATGTGGGTCCCACATTTATATTGAAATAAAAAAAATATAAAAATAACTAAATAAA | 53.1 |
| Fz-15-7A  (MF141737) | 1043-1218 | 175 | 92 | AATAGATTATATTAAAGAGAGAAGGGCCGCGCAGCGGCAATAACATGAGTAATTTTGCTTCTTGAAAACAAGAAGGAATGAAATGAAAAAAAGAAATAAAAAAAAAAAATTGTAATATAAATGTGGGTCCCACATTTATATTGAAATAAAAAAAATATAAAAATAACTAAATAAA | 53.4 |
| Fz-15-10A  (MF141738) | 1033-1220 | 187 | 92 | AAGACAAGAAAGAATGAAATGAAAAAAAAAACAAAAACATGAGCCTTCTACTATTTTATTTATGGAATAAGTGCCGCGCAGCGGCATTTAAAAAAATAAAAATAAAAAAGAAGTATAAAAATATATTTATTATGCCTTTCTAAAACGACGACGTATTGGAAAAGTGTAAATGGGACCAAAATGTAAA | 49.1 |
| Fz-16-7A  (MF141739) | 1043-1216 | 173 | 91 | AATAGATTATATTAAAGAGAGAAGGGCCGCGCAGCGGCAATAACATGAGTAATTTTGCTTCTTGAAAACAAGAAGGAATGAAATGAAAAAAAGAAATAAAAAAAAAAATGTAATATAAATGTGGGTCCCACATTTATATTGAAATAAAAAAAATATAAAAATAACTAAATAAA | 52.6 |
| Hmg-14-1A  (MF141740) | 1034-1156 | 122 | 73 | AAAACAAGAAAGAATGAAATAAAAAAAGAAAAAGGAAAAACGTGAGCCTTCTATTAATTTAAGAAAGAAAGTGCCGCGCAGCGGCATTCAAAAAAAAATATAAATATAAAAAAAGAAAAAAA | 59.8 |
| Hmg-15-6A  (MF141741) | 1033-1220 | 187 | 92 | AAGACAAGAAAGAATGAAATGAAAAAAAAAACAAAAACATGAGCCTTCTACTATTTTATTTGCGGAATAAGTGCCGCGCAGCGGCATTAACAAAAATAAAAATAAAAAAAAAAGTATAAAATATATTTATTATGTCTTTCTAAAACGACGACGTATTGGAAAAGTGTAAATGGGACCAAAATGTAAA | 49.1 |
| SG-14-23A  (MF141742) | 1034-1219 | 186 | 91 | AAGACAAGAAAGAATGAAATGAAAAAAAAACAAAAACATGAGCCTTCTACTATTTTATTTGCGGAATAAGTGCCGCGCAGCGGCATTAACAAAAATAAAAATAAAAAAAAAAGTATAAAATATATTTATTATGTCTTTCTAAAACGACGACGTATTGGAAAAGTGTAAATGGGACCAAAATGTAAA | 48.9 |
| SG-15-11A  (MF141743) | 1034-1220 | 187 | 92 | AAGACAAGAAAGAATGAAATGAAAAAAAAAACAAAAACATGAGCCTTCTACTATTTTATTTGCGGAATAAGTGCCGCGCAGCGGCATTAACAAAAATAAAAATAAAAAAAAAAGTATAAAATATATTTATTATGTCTTTCTAAAACGACGACGTATTGGAAAAGTGTAAATGGGACCAAAATGTAAA | 49.1 |
| SG-16-5A  (MF141744) | 1033-1220 | 187 | 92 | AAGACAAGAAAGAATGAAATGAAAAAAAAAACAAAAACATGAGCCTTCTACTATTTTATTTATGGAATAAGTGCCGCGCAGCGGCATTTAAAAAAATAAAAATAAAAAAGAAGTATAAAAATATATTTATTATGCCTTTCTAAAACGACGACGTATTGGAAAAGTGTAAATGGGACCAAAATGTAAA | 49.1 |
